# Protecting cows in small holder farms in East Africa from tsetse flies by mimicking the odor profile of a non-host bovid

**DOI:** 10.1371/journal.pntd.0005977

**Published:** 2017-10-17

**Authors:** Rajinder K. Saini, Benedict O. Orindi, Norber Mbahin, John A. Andoke, Peter N. Muasa, David M. Mbuvi, Caroline M. Muya, John A. Pickett, Christian W. Borgemeister

**Affiliations:** 1 International Centre of Insect Physiology and Ecology (*icipe*), Nairobi, Kenya; 2 Pestinix-International Pest & Vector Control Specialists, Nairobi, Kenya; 3 African Population and Health Research Center (APHRC), Nairobi, Kenya; 4 Africa Union–Interafrican Bureau for Animal Resources (AU-IBAR), Nairobi, Kenya; 5 Rothamsted Research, Harpenden, Herts., AL, United Kingdom; 6 Center for Development Research (ZEF), University of Bonn, Bonn, Germany; Institut de recherche pour le developpement, FRANCE

## Abstract

**Background:**

For the first time, differential attraction of pathogen vectors to vertebrate animals is investigated for novel repellents which when applied to preferred host animals turn them into non-hosts thereby providing a new paradigm for innovative vector control. For effectively controlling tsetse flies (*Glossina* spp.), vectors of African trypanosomosis, causing nagana, repellents more powerful than plant derived, from a non-host animal the waterbuck, *Kobus ellipsiprymnus defassa*, have recently been identified. Here we investigate these repellents in the field to protect cattle from nagana by making cattle as unattractive as the buck.

**Methodology/Principal findings:**

To dispense the waterbuck repellents comprising guaiacol, geranylacetone, pentanoic acid and δ-octalactone, (patent application) we developed an innovative collar-mounted release system for individual cattle. We tested protecting cattle, under natural tsetse challenge, from tsetse transmitted nagana in a large field trial comprising 1,100 cattle with repellent collars in Kenya for 24 months. The collars provided substantial protection to livestock from trypanosome infection by reducing disease levels >80%. Protected cattle were healthier, showed significantly reduced disease levels, higher packed cell volume and significantly increased weight. Collars >60% reduced trypanocide use, 72.7% increase in ownership of oxen per household and enhanced traction power (protected animals ploughed 66% more land than unprotected). Land under cultivation increased by 73.4%. Increase in traction power of protected animals reduced by 69.1% acres tilled by hand per household per ploughing season. Improved food security and household income from very high acceptance of collars (99%) motivated the farmers to form a registered community based organization promoting collars for integrated tsetse control and their commercialization.

**Conclusion/Significance:**

Clear demonstration that repellents from un-preferred hosts prevent contact between host and vector, thereby preventing disease transmission: a new paradigm for vector control. Evidence that deploying water buck repellents converts cattle into non-hosts for tsetse flies—*‘cows in waterbuck clothing’*.

## Author summary

We investigated the potential of non-host odors from un-preferred animals, i.e. not fed upon, related to cattle, the waterbuck (*Kobus ellipsiprymnus defassa*) which are common in tsetse habitats for their efficacy to protect cattle from tsetse flies that transmit nagana to cattle in Africa. The identified waterbuck repellent compounds comprising of geranylacetone, pentanoic acid, guaiacol and δ-octalactone (patent application) were dispensed on cattle (hosts) using innovative repellent dispensers that individual cattle wear encircling their necks. This study, which is the first of its kind, shows that the waterbuck repellent compounds can provide substantial protection to cattle from trypanosome infections by reducing disease levels >80% in protected animals. Thus, by mimicking the odor profile of waterbucks, ‘*cows in waterbuck clothing’* are essentially turned into non-hosts for tsetse flies and thus provide a new paradigm for innovative vector control. In our trial, protection of cattle with the repellent collars resulted in considerably improved food security as a result of significant reduction in trypanocide use, improved health of the protected animals which enhanced traction power with more land being brought under cultivation. Farmers’ perceptions of the repellent collars are very positive and socioeconomic studies indicate that adoption potential is extremely high.

## Introduction

Infectious diseases affecting livestock and human health that involve vector borne pathogens are a global problem and contribute directly to food insecurity and poverty, especially in sub-Saharan African (SSA) countries. This situation is further exacerbated by the emergence of new vector borne diseases, and difficulties with the control of old vector borne diseases such as malaria and other neglected tropical diseases (NTDs) e.g. trypanosomosis, generating urgent demands for new effective tools and strategies for the control of these pathogen vectors. Vector behavior modification to prevent an arthropod entering a space occupied by a potential livestock or human host to reduce encounters between the host and vectors thereby eliminating or reducing the risk of pathogen transmission is one avenue which may lead to generation of effective chemicals and novel products for disease /vector control. However, a better understanding of the host orientated behavior of vectors is needed for innovative product development to enhance vector control. Host oriented behavior of disease vectors such as mosquitoes, ticks and tsetse flies (*Glossina* species) is not only dependent on kariomones from preferred vertebrate hosts but increasing evidence indicates that these vectors also avoid unsuitable or un-preferred animals by means of distinct chemical odors [1]. Thus, the differential attraction of biting insects during the host location process involves the detection of ‘non-host’ compounds or repellents as well as ‘host’ attractants, especially during the discrimination between different hosts [1–5] and even individuals within a host species. By understanding these complex interactions new novel semiochemicals with potential control applications can be uncovered.

**Fig 1:**
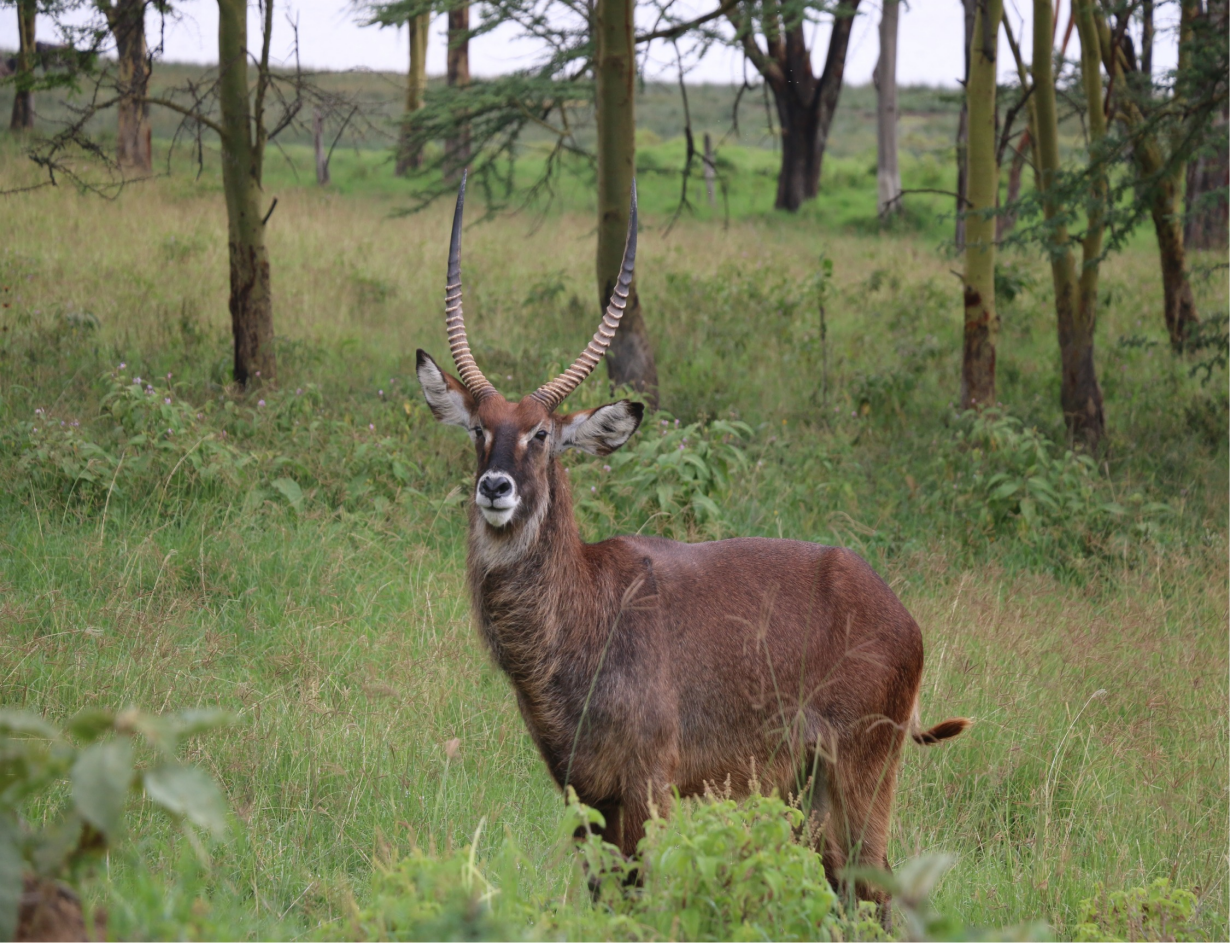
Waterbuck *Kobus ellipsiprymnus defassa* Rüppel (Bovidae). (Picture R.K. Saini).

Here we investigate a new class of repellents from non-preferred vertebrate animals that are highly active due to their ecological role in non-host avoidance. These repellents have been identified from waterbuck *Kobus ellipsiprymnus defassa* Rüppel (Bovidae) (Fig 1) which are present in tsetse habitats but are not fed upon even though they belong to the same family as the preferred hosts (cattle) common in tsetse habitats for its efficacy to protect cattle from tsetse flies vectors of African trypanosomosis causing nagana in cattle in Africa [4]. *Glossina* spp. feed exclusively on vertebrate blood and in doing so transmit African animal trypanosomosis (nagana) in cattle (Bovidae), and sleeping sickness in humans. Nagana is the major constraint on milk and meat production and animal traction in small holder animal husbandry in SSA, causing annual losses to the livestock and crop production sectors of >4.7 billion US$ [6]. In fact, tsetse and trypanosomiasis influence where people live, how they manage their livestock and how intensive and productive crop agriculture can be practiced [7].

### The waterbuck repellent compounds (WRC)

**Fig 2:**
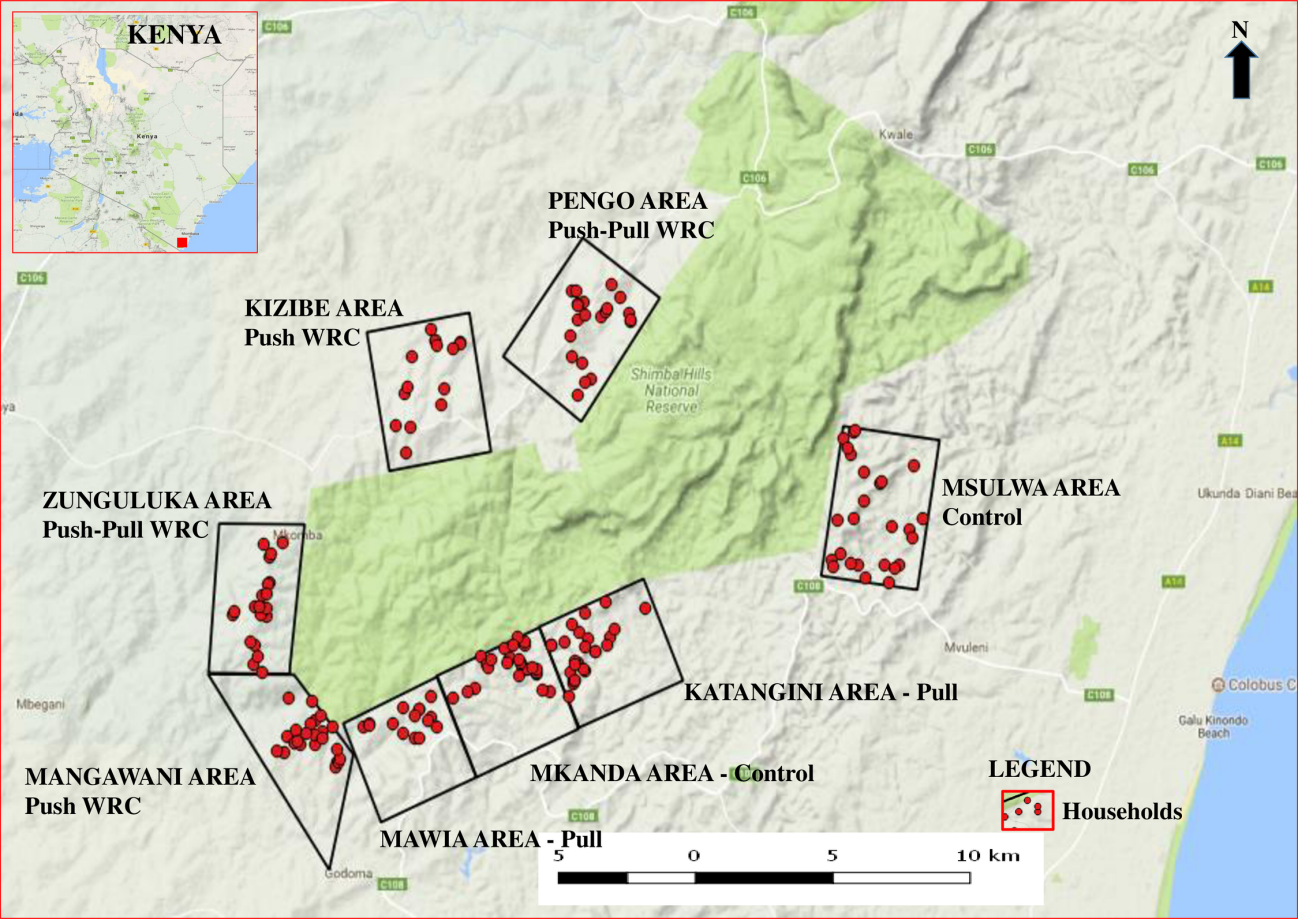
Map showing experimental sites in SHGR and location of households which provided cattle for the trial.

Many semiochemicals have recently been identified that are thought to be involved in differential attraction of biting flies to individual vertebrate hosts but most work has focused on malaria mosquitoes [1]. The natural differential attractiveness of certain vertebrate hosts by tsetse flies (*morsitans* group of flies) has been known for a long time and recent studies in our laboratory have started to elucidate the semiochemical basis that savannah tsetse flies use to avoid non-preferred animals such as waterbuck [3,4, 8–10]. Our earlier study on the responses of savannah tsetse (*G*. *m*. *morsitans* and *G*. *pallidipes*) to feeding membranes with waterbuck sebum exhibited less probing and feeding compared to control membranes suggesting the involvement of chemical cues in mediating aversion by these ectoparasites to this bovid [8]. Analysis by combined gas chromatography and electroantennographic detection (GC-EAD) analysis and coupled mass-spectrometry (GC-MS) led to the identification of a series of fifteen compounds specific to waterbuck, including straight chain carboxylic acids, ketones, phenols and a lactone, δ-octalactone [3, 4, 8–10]. Recently, the effect of different blends of these compounds on catches of *G*. *pallidipes* (Austen) in attractant-baited NG2G traps [11] were evaluated in the field through a series of subtractive assays to determine the contribution of different classes and constituents to waterbuck odor repellency [4]. While, each of the four classes of odor compounds contributes to overall repellency, a 5-component blend of compounds selected from each class based on results obtained in subtractive assays (guaiacol, geranylacetone, hexanoic and pentanoic acid and δ-octalactone,) showed substantial reduction in fly catches (84%) relative to the baited control and comparable to the 15-component blend [3,4]. In separate experiments, involving an ox tethered in the middle of an incomplete ring of electric screens, to determine feeding efficiency of the flies [10] in the presence or absence of 15-component or 5-component blends, comparable levels in the reduction of fed flies (94 and 95%, respectively) were obtained with the two blends [4]. Since a 4-component blend comprising of geranylacetone, guaiacol, pentanoic acid and δ-octalactone–Patent Application: KE 771, PCT/KE/2014/000037, KE/P/2013/001888, US15/016,303 [3,4], representing each of the said four classes of odor compounds also reduced fly catches by 84%, this blend herein referred to as the waterbuck-repellent compounds (WRC), was evaluated for its repellency on the host animals under natural tsetse challenge. This 24-month large-scale field evaluation trial was conducted near the Shimba Hills Game Reserve (SHGR) in Kubo Division, Kwale County in the coastal area of Kenya (latitude 3°3'S and 4°45'S south and longitudes 38°31'E and 39°831'E) [12] involving > 1,100 cattle (Fig 2). Prior to the initiation of the large-scale field trial, baseline parasitological and entomological surveys were undertaken to determine the prevalence of bovine trypanosomosis and the apparent densities of tsetse flies in the study sites area, the results of which together with the diagnostic techniques employed have been published earlier [12]. This area where farmers practice subsistence farming is infested predominately with *G*. *pallidipes* (apparent density of 30 flies/trap/day), *G*. *austeni* (0.8 flies/trap/day) and *G*. *brevipalpalis* (0.4 flies/trap/day) with trypanosome prevalence in cattle of 33.9% [12]. In these sites both *T*. *congolense* (61.1%), *T*. *vivax* (38.9%) were found in the cattle that tested positive for trypanosomes [12].

**Fig 3:**
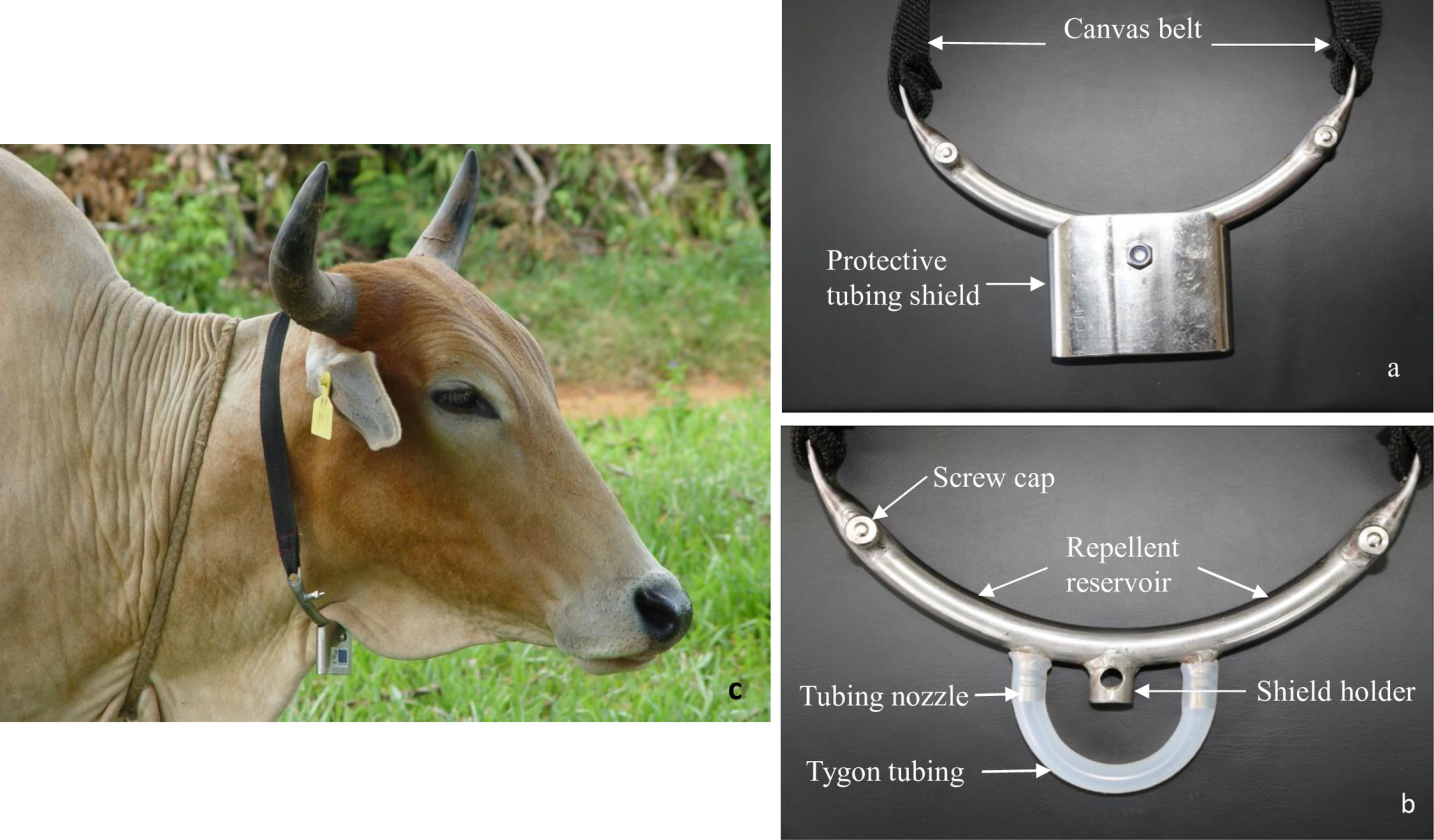
Repellent dispenser developed to deliver waterbuck repellent compounds for cattle. (a) Dispenser with protective shield and (b) without protective shield to show tubing from which the repellent compounds are released and (c) a cow with a repellent collar.

In order to minimize/prevent contact between host and vector to prevent disease transmission to livestock, dispensing non-host odors on a host animal is challenging. The repellent compounds not only need to be released at a constant rate from the cattle to turn them effectively into tsetse non-hosts but any device should be compatible with the low level of resources available in SSA to pastoralists/ small scale farmers for whom nagana is a major constraint. In order to dispense the water buck repellent compounds on cattle we developed innovative repellent collars which are described in Fig 3A, 3B & 3C. Essentially, the WRC release device comprises of a reservoir into which the WRC is injected and silicon tubing from which it diffuses at constant release rate. It is tied around the neck of cattle using canvas belts (Fig 3C).

**Fig 4:**
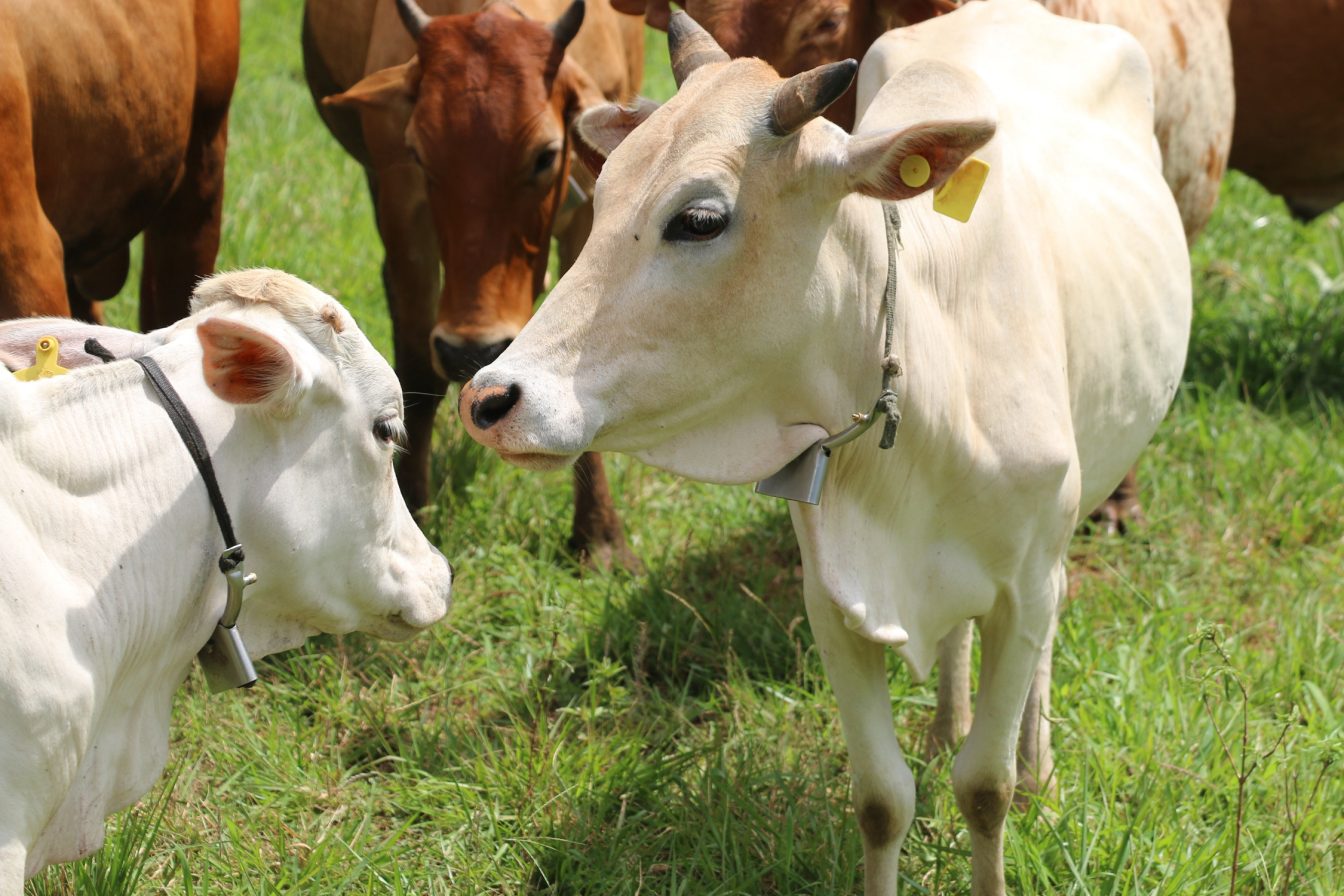
Cattle with repellent collars grazing in bush with natural tsetse challenge. (Picture R.K. Saini).

The 4-component WRC comprised of guaiacol, geranylacetone, pentanoic acid and δ-octalactone [3,4] blended roughly in a ratio of 2:1:3:3 respectively as found naturally on the waterbuck odor [9]. In this study, the main objective was to investigate the efficacy, under natural tsetse challenge of this new class of repellents for their efficacy to protect cattle from tsetse flies that transmit nagana to cattle (Fig 4).

**Fig 5:**
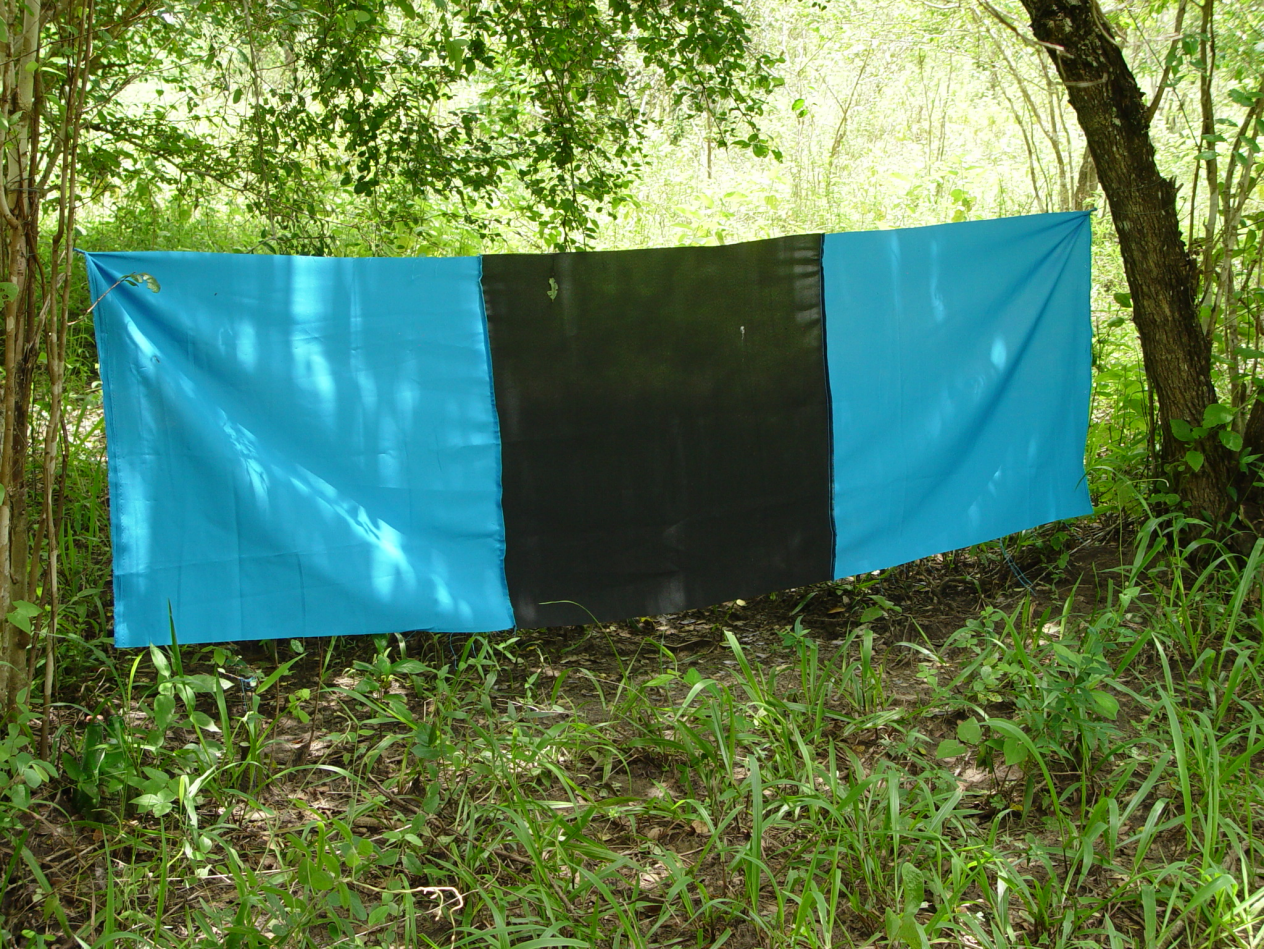
Target used to attract and kill the flies (pull). (Picture R.K. Saini).

We also tested the repellents in a push-pull strategy demonstrated only previously in agricultural cropping systems [13] to determine if any synergistic effects could be obtained to enhance disease and fly suppression by simultaneously deploying repellent collars to push the flies away and targets baited with cow urine and acetone [11] to attract and kill the flies (with the fly killing agent deltamethrin) as pull (Fig 5) Vector and disease data collection was accompanied by an independent socio-economic study on the perceptions of pastoralists/ farmers on the efficacy of the repellent collars, their impact on trypanocide drug use, animal traction power and their willingness to invest in the novel technology. The treatments included: (i) push-pull-WRC, i.e. cattle with WRC collars grazing in an area with baited targets; (ii) pull, i.e. unprotected cattle grazing in an area with baited targets; (iii) push-WRC, i.e. cattle protected with WRC collars and grazing in an area without targets; and (iv) control, i.e. unprotected cattle grazing in an area without targets (see Table 1).

**Table 1:** Randomized four treatment regimes for the eight trial sites*.

**Treatment**	**Sites**	**No. of farmers**	**No. of cattle**	**No. of targets**
**Push-Pull WRC** (*Cattle with WRC collars grazing in an area having targets*)	Pengo	26	111	64
	Zunguluka	18	117	78
**Pull** (*unprotected cattle grazing in an area with baited targets*)	Mawia	20	155	89
	Katangini	31	176	72
**Push–WRC** (*cattle protected with WRC collars and grazing in an area without targets*)	Mangawani	26	141	
	Kizibe	20	131	
**Control** (*unprotected cattle grazing in an area without targets*)	Mkanda	20	129	
	Msulwa	19	145	
**Total**		**180**	**1,105**	**303**

* On the outskirts of Shimba Hills Game Reserve, Kenya Coast

Here we present evidence of how by deploying the water buck repellent compounds on cattle we can essentially turn them into non-hosts for tsetse flies—*‘cows in waterbuck clothing’* and use these novel repellents for livestock protection.

## Materials and methods

### Intervention area

Fig 2 shows the intervention area on the outskirts of Shimba Hills Game Reserve (SHGR) in Kubo Division, Kwale County in the coastal area of Kenya (latitude 3°3'S and 4°45'S south and longitudes 38°31'E and 39°831'E) [12]. In this area, the tsetse repellent collars impregnated with WRC were evaluated for their efficacy in providing protection to cattle (host animals) from nagana under natural tsetse challenge in a 24-month large scale trail which was conducted between July 2011 (when intervention started) and June 2013. This area where farmers practice subsistence farming is infested predominately with *G*. *pallidipes* (apparent density of 30 flies/trap/day), *G*. *austeni* (0.8 flies/trap/day) and *G*. *brevipalpalis* (0.4 flies/trap/day) with trypanosome prevalence in cattle of 33.9% [12]. Since *G*. *pallidipes* is the major tsetse species caught in the intervention sites all results herein reported pertain to this species. In these sites both *T*. *congolense (54*.*6%)* and *T*. *vivax* (44.1%) are found in the cattle. Mixed infections of the two trypanosomes are found in about 1.3% of infected cattle. SHGR is endowed with variety of wild animals and is a good breeding environment for tsetse flies.

### Site selection

Eight sites: Pengo, Zunguluka, Mawia, Katangini, Mangawani, Kizibe, Mkanda, and Msulwa (Table 1; Fig 2) were randomly selected for the trials. These sites were selected based on the following criteria: (i) they were of approximately the same size (each of which was about 16 km^2^ (15.63±0.52 km^2^), with no significant difference in size between the selected sites); (ii) close to SHGR boundary which is a high tsetse challenge area; (iii) farmers’ were willing to participate in the trials and committed to provide sufficient cattle for at least 12 months and (iv) the sites were easily accessible during the rainy season and with high tsetse and trypanosomosis challenge. The sites were named after the main administrative locations in which they were located but did not necessarily follow the boundaries.

### Selection of farmers and stakeholder meetings

Selection of farmers for the trials was a difficult exercise and for this, 12 different stakeholder meetings were held with over 467 different farmers/ pastoralists involved. In these meetings, officials from the Veterinary Department and the Livestock Production Department of the Kenyan Ministry of Livestock Development were always present. Approximately 20 farmers were selected from each site by drawing lots, totaling 180 farmers and 1,105 head of cattle (Table 1; Fig 2).

### Baseline parasitological and entomological surveys and trial sites

Prior to the initiation of the large-scale field trial, baseline parasitological and entomological surveys were undertaken to determine the prevalence of bovine trypanosomosis and the apparent densities of tsetse flies in the study sites area, the results of which together with the diagnostic techniques employed have been published earlier [12].

### Treatment regimes

The treatments included: (i) push, i.e. cattle protected with WRC collars (see description below) and grazing in an area without targets (ii) pull, i.e. unprotected cattle grazing in an area with baited targets, (screens with blue-black-blue polyester panels each 1.0m^2^ in size with black middle panel impregnated with deltamethrin (4.5g/kg) and a UV-protector to add to the lifetime of the colored fabric supplied by Vestergaard Frandsen Ltd. (Fig 5). These targets were baited with cow urine (~1,000mg/h) and acetone (~ 500mg/h) [11] and replaced every 9 months during the trial period (the suppliers recommend that the targets should be replaced every 12 months); (iii) push-pull, i.e. cattle with WRC collars grazing in an area with baited targets and (iv) control, i.e. unprotected cattle grazing in an area without targets. 4–5 odor baited targets per km^2^ depending on the habitat were deployed as pull treatment as at this density they have been estimated to be sufficient to control *morsitans* group of flies [14]. All cattle were kept under traditional local farmer management and were brought to a cattle crush in each of the sites for sampling every month.

The rational for the push-pull treatment involved the WRC collars (spatial repellents) to ‘push’ the flies away and an insecticide impregnated target to ‘pull’ and kill the flies (removal trapping). A similar push-pull system was proposed for haematophagous insects that bite livestock [15] and recently for control of malaria mosquitoes [16]. Treatments were randomized in the eight trial sites and involved 180 farmers, 1,105 cattle and 303 baited targets the distribution of which per site is shown in Table 1.

Prior to the start of the trial, all animals were blanket treated with diminazene diaceturate (Veriben manufactured in France by Ceva Sante Animale) at doses of 3.5mg/kg, by intramuscular injection, body weight being estimated by weighing bands. This is a curative treatment with no lasting protection against re-infection.

### Repellent dispensers

Fig 3, shows a schematic drawing of a prototype device used to dispense the WRC on the host animals (*icipe* patent pending). These bow-shaped devices were made of stainless steel with a repellent reservoir (24cm in length and internal diameter of 1.27cm) into which the repellent blend was injected through two nozzles at each end (to protect from any airlock) which were later closed by screw caps. The reservoir in the middle was connected through two nipples to tygon silicon tubing (9cm long, 0.635cm internal diameter, 0.953cm external diameter and wall thickness of 0.159cm; Cole Parmer International) through which the WRC were released into the atmosphere. A 9.0cm long tygon tubing was used because in our previous experiment

4-methylguaiacol at a release rate of about 9.0mg/hr showed maximum reduction of *G*. *pallidipes* [17]. The tubing in each dispenser was covered by a protective shield to minimize damage during grazing. Both ends of the reservoir were pressed to provide for holes for attachment of the canvas belts with which they were tied around the neck of the cattle (Figs 3 and 4). The bow-shape of the dispenser enabled it to fit comfortably around the neck of the animal. Each reservoir was filled with 10ml of the WRC blend (see composition and ratio below) to last more than a month, though when filled at its maximum capacity (34.5ml) and provided there was no leakage the dispenser could last up to approximately 4.5 months at a release rate of 10.484±0.107mg hr^1^ as determined by weighing daily over a one-month period (N = 10 dispensers). However, the relative rates of diffusion of different constituents from the tygon tubing and their evaporation from the surface would be expected to be affected by their structures, sizes and relative amounts in different blends. At each monthly sampling, dispensers that were not damaged were replenished with 7.5ml of the WRC to cater for the weight loss in a month. Damage to dispensers was also recorded during sampling and damaged dispensers were repaired and re-filled. Lost dispensers were replaced.

### Composition of WRC (waterbuck repellent mixture)

The 4-component WRC used in the repellent dispensers throughout the trials comprised of guaiacol, geranylacetone, pentanoic acid and δ-octalactone (patent application: KE 771, PCT/KE/2014/000037, KE/P/2013/001888, US15/016,303) [3, 4] blended approximately in the ratio 2:1:3:3, respectively, as found naturally in the waterbuck odor [10]. Chemicals were purchased from Sigma-Aldrich and ChemSampCo, LLC. The purity of each chemical was 99%.

## Sampling

### Target servicing

Targets (Fig 5) were serviced monthly by topping up the attractants (cow urine and acetone) and clearing the vegetation around them. The targets which were torn by wind or destroyed by cattle were either sown or replaced if completely damaged. All targets were replaced after nine months due to heat discoloration. Some targets were also subject to stealing or vandalism or destruction by fire and these were replaced whenever noticed (in the first year after 3 months (October 2011) at push-pull sites of Pengo and Zunguluka and at pull sites of Mawia and Katangini 15, 5, 6 and 4 targets were lost due to fire while in Mawia and Katangini 10 and 4 targets were lost due to theft in the first year while in Zunguluka 15 and Mawia 10 targets were respectively lost in the 2^nd^ year of the trial).

### Animal sampling and parasitological surveys

All recruited animals were ear-tagged and blood-sampled once every month. For this they were restrained in a crush, their ear veins pricked with a lancet and blood collected using a pair of heparinised micro-haematocrit centrifuge capillary tubes. The capillary tubes were sealed with “Cristaseal” (Hawksley) and centrifuged immediately in a micro-haematocrit centrifuge for 5 min at 9,000 rpm and presence of trypanosomes in the blood was determined using the buffy-coat technique (BCT) under field conditions [18]. Trypanosomes were identified by species based on their morphological and motility characteristics observed on wet preparations of the buffy coat. Pack Cell Volume (PCV) was also determined for each animal using a PCV reader (Hawskley Ltd., UK).

The BCT technique was used as the method is simple, inexpensive, field based and gives immediate results which the farmers want for their cattle to be treated immediately if tested positive. In our trial, resource limitations and the very large sample size (>1,100 blood samples every month) did not permit more sensitive molecular, time consuming and expensive tests in our laboratory situated > 500km away from the field sites. Furthermore, to improve the diagnostic ability of the trypanosomosis screening practices and reliability of the BCT technique, as suggested by Maudlin et al. [19] animals were sampled very early in the morning by collecting venous blood from the peripheral ear veins and minimizing the time between blood collection and screening and by increasing the sensitivity of the technique by concentrating the trypanosomes by microhaematocrit centrifugation in the field. Furthermore, the diagnostic sensitivity of the method also depends on the examiner’s experience which in our case was more than 15 years on routine bases for the technical staff involved in parasitological diagnosis.

All animals found positive with trypanosomes or having a PCV of <24% were treated with Veriben at 7mg/kg body weight, administered intramuscularly. Weight of each animal sampled (using weight measurements tape calibrated for cattle and pigs [Rondo, UK] and by measuring the girth of the animal) was also recorded and wounds, tick-borne infections and worm infestations that were observed or presented by cattle owners were managed appropriately.

### Entomological monitoring

Ten NGU traps [11] baited with acetone and cow urine in each site were used to monitor tsetse and other biting flies’ populations monthly. Traps were checked each day for three consecutive days and the apparent density calculated as the mean number of flies captured per trap per day (FTD). Records were kept of the number, species and sex of tsetse captured in each trap.

### Social economic evaluation of the repellent collars

The impacts of the repellent collars in Shimba Hills were evaluated. For this households were sampled as per the treatment regimes adopted for the evaluation of the WRC (Table 1; Fig 2). Interviewer-administered questionnaires (see S1 & S2 Files), which were pre-tested, were applied for the quantitative individual farmer sample survey (N = 178) to elicit information on: perceptions of the farmers on the efficacy of the repellent collars *vis-à-vis* other tsetse control technologies, changes in grazing patterns, oxen owned, traction power and land cultivated for crop production. Perceptions of the livestock farmers on trypanocide use and costs of treatments were also assessed. Willingness of farmers to invest in the tsetse repellent collars was also assessed.

### Data analysis

Statistical analyses were performed using SAS [20]. For the trypanosomosis prevalence data, a generalized estimating equations (GEE) model, assuming autoregressive working correlation, was fitted to test whether over time the proportion positive for infection with trypanosomes varied significantly among the treatment groups. The covariates included in the GEE model were treatment, skin color of the animal, sex of the animal (male = 0, female = 1), time (i.e. month of observation) and treatment-by-time interaction. To investigate whether PCV scores and cattle body weights also varied significantly among the four treatment groups over time, a linear mixed effect (LMM) model was fitted separately for PCV (with the covariates: treatment, weight, sex, age of the animal (adult = 0, calf = 1), time and time-treatment interaction) and body weight (with the covariates: treatment, animal age, skin color, time and time-treatment interaction term) [21]. The number of *G*. *pallidipes* flies caught in various WRC treatments were compared using a negative binomial model. The factors included in the model were treatment and time (i.e. month of sampling).

Socio-economic data were analyzed using non-parametric tests. Differences between treatments were examined using Kruskal-Wallis test and Wilcoxon rank sum test was used to compare differences within treatments. In cases where observations between treatments differed significantly, a post-hoc analysis was carried out using Mann-Whitney U test with a Bonferroni adjustment and counter checked with results from Dunn’s test, to determine which treatment differed significantly from another. Parameters N and n within parenthesis are distinctively used to refer to the whole sample population and a subset of the sample population, respectively. All tests were performed at 5% significance level.

## Ethics statement

This study was undertaken in adherence to experimental guidelines and procedures approved by ICIPE’s Institutional Animal Care and Use Committee (IACUC, Ref: *icipe*/2009/222240). These IACUC regulations conformed to national guidelines provided by the Kenya Veterinary Association. All cattle in the trial were treated free of charge in case they became sick.

## Results

### Effect of repellent collars on trypanosomiaisis incidence

**Fig 6:**
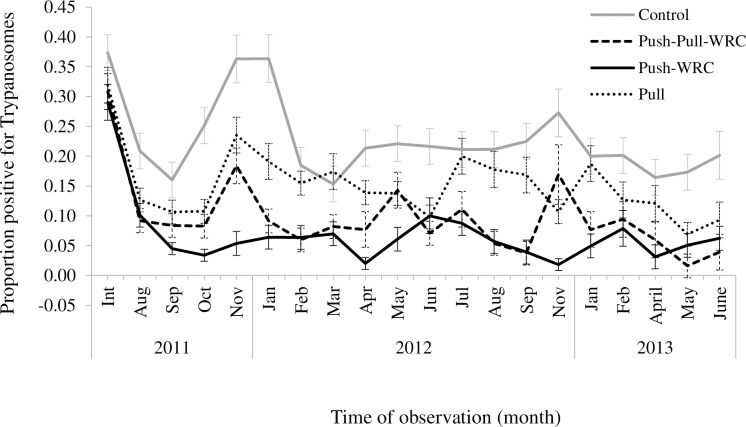
Monthly trypanosome infection incidence in cattle with various tsetse repellent (WRC) treatments over the trial period (Int–start of intervention).

Using BCT [18], we observed a general decrease in the proportion of animals positive for trypanosomosis over time (Fig 6), with a greater proportion of positive tested animals in the un-protected group without repellent collars (control) than in push-pull-WRC, push-WRC, and pull throughout the entire study period.

The Generalized estimating equation (GEE) analysis (Table 2) revealed a significant ‘time-by-treatment’ interaction effect (Chi Sq = 29.93, df = 3, P<0.0001), implying that on average, at least any two of the four treatments varied significantly over time. Compared to animals without repellent collars and no targets around (control), infection over time among animals protected with repellent collars and in an area with (push-pull-WRC) and without targets (push-WRC) were significantly lower (push-pull-WRC: OR = 0.94, 95% CI 0.91–0.97; push-WRC: OR = 0.93, 95% CI 0.90–0.97), but not significantly so for un-protected animals in an area with targets (pull) (OR = 0.99, 95% CI 0.97–1.01). Compared to pull, the animals in the push-pull-WRC and push-WRC groups were significantly less infected (push-pull-WRC: OR = 0.95, 95% CI 0.91–0.98; push-WRC: OR = 0.94, 95% CI 0.91–0.98). However, there was no significant difference in infection by trypanosomes between animals in the push-pull-WRC and push-WRC treatment groups (P = 0.8614). Overall, animals with repellent collars showed on average 80% lower levels of infection (86% and 80% reduction in the push-WRC and push-pull-WRC treatments, respectively from baseline), compared to only about 60% reduction in animals without repellent collars and where only targets (pull) were present and about 50% with the control animals which essentially were therapeutic control as they were treated with trypanocides when found to be infected. Thus, substantial protection from trypanosome infections under natural tsetse challenge was achieved in animals with repellent collars. Skin color (Chi Sq = 24.99, df = 6, P = 0.0003) and sex (Chi Sq = 31.48, df = 1, P<0.0001) of the animals were also significantly associated with trypanosome infections, with female animals having lower infection by trypanosomes than males. Compared to black, the white and brownish red colored animals had significantly lower infection. Among the cattle that tested positive for trypanosomes 54.6%, 44.1% and 1.3% were infected by *T*. *congolense*, *T*. *vivax* or both species, respectively.

**Table 2:** Trypanosome infection in cattle with waterbuck repellent compounds (WRC). Estimates obtained from the generalized estimating equations model. The 95% Confidence Intervals (CI) were constructed using empirically corrected standard errors.

Variable		Odds Ratio	95% CI		P value
*Treatment*					
	Control	1.00			
	push-pull-WRC	0.53	0.40	0.68	<0.0001
	push-WRC	0.38	0.29	0.50	<0.0001
	pull	0.68	0.54	0.86	0.0014
*Skin Color*					
	Black	1.00			
	White	0.78	0.63	0.96	0.0208
	Brown	0.85	0.71	1.03	0.0944
	Brownish red	0.58	0.44	0.77	0.0002
	Cream	1.00	0.80	1.26	0.9710
	Grey	0.87	0.70	1.09	0.2262
	Others	1.04	0.85	1.28	0.6844
*Sex*					
	Male	1.00			
	Female	0.71	0.63	0.80	< .0001
*Time*		0.97	0.96	0.99	0.0008
Time*Treatments					
	Time*Control	1.00			
	Time*push-pull-WRC	0.94	0.91	0.97	0.0004
	Time*push-WRC	0.93	0.90	0.97	0.0005
	Time*pull	0.99	0.97	1.01	0.3876

### Effect of repellent collars on packed cell volume

In all four treatment groups, mean PCV values increased sharply soon after the intervention, followed by an equally sharp drop, especially in the control group. Throughout the entire study period, PCV scores were highest in animals with collars in the push-WRC group. To investigate any differences in the PCV scores across treatments, we used a linear mixed model with the same mean structure as that for the GEE less skin color, but with the weight, sex, time and age of the animal adjusted for (Table 3). Scores differed significantly over time among the treatment groups: Compared to the control and after adjusting for the other factors (i.e. weight, sex, age and time) animals in the push-pull-WRC (P = 0.0229), push-WRC (P<0.0001) and pull (P = 0.0046) groups had on average significantly higher PCV scores over time. Among the treatment groups, PCV scores differed significantly between push-pull-WRC and push-WRC (P = 0.0042), push-WRC and pull (P = 0.0006), but not between push-pull-WRC and pull (P = 0.9748). Weight, sex and age of the animal were also significantly associated with PCV scores (Table 3).

**Table 3:** Packed cell volume (PCV) scores with various waterbuck repellent compounds (WRC) treatments. Parameter estimates and standard errors obtained from linear mixed models for the association between PCV scores and various WRC treatments over the trial period.

Variable		Estimate	Standard Error	P value
Intercept		25.19	0.34	<0.0001
Treatments				
	Control	0.00		
	push-pull-WRC	0.33	0.32	0.2965
	push-WRC	0.69	0.30	0.0191
	pull	0.38	0.28	0.1710
Weight		0.01	0.00	<0.0001
Sex	Male	0.00		
	Female	0.89	0.18	<0.0001
Age	Adult	0.00		
	Calf	1.22	0.29	<0.0001
Time		-0.14	0.02	<0.0001
Time*Treatment				
	Time*Control	0.00		
	Time*push-pull-WRC	0.06	0.03	0.0229
	Time*push-WRC	0.14	0.02	<0.0001
	Time*pull	0.06	0.02	0.0046

### Effect of repellent collars on weight of the animals

Compared to the control and after adjusting for the other covariates (i.e. sex, age, skin color and time), over time the weight of animals in the other treatment groups were on average significantly higher (linear mixed model; Table 4). Animals with repellent collars (in push-WRC and push-pull-WRC groups) had significantly higher weight gain over time compared to unprotected animals (control) or those with targets alone (pull-WRC; P = 0.0172). Even within animals protected with repellent collars those in the push-pull treatment had significantly higher weight gain than push (P = 0.0104). Animal color was associated with body weight, with cream colored animals being significantly heavier than black ones (Table 4).

**Table 4:** Animal weights with waterbuck repellent compounds (WRC). Parameter estimates and standard errors obtained from linear mixed model relating animal weight with waterbuck repellent compounds (WRC) treatments over the trial period.

Variable		Estimate	Standard Error	P value
Intercept		178.12	3.85	<0.0001
Treatments				
	Control	0.00		
	push-pull-WRC	24.12	3.97	<0.0001
	push-WRC	15.80	3.74	<0.0001
	pull	15.35	3.26	<0.0001
Sex	Male			
	Female	-6.98	2.25	0.0020
Age	Adult	0.00		
	Calf	-51.20	3.11	<0.0001
Skin Color	Black	0.00		
	White	-4.30	4.14	0.2993
	Brown	4.51	3.74	0.2275
	Brownish red	5.13	5.14	0.3185
	Cream	19.77	5.36	0.0002
	Grey	5.76	4.74	0.2247
	Others	-3.70	3.95	0.3486
Time		2.33	0.09	<0.0001
Time*Treatment				
	Time*Control	0.00		
	Time*push-pull-WRC	0.70	0.15	<0.0001
	Time*push-WRC	1.10	0.13	<0.0001
	Time*pull	0.33	0.13	0.0080

### Effect of tsetse repellent (WRC) treatments on apparent densities of *G*. *pallidipes*

**Fig 7:**
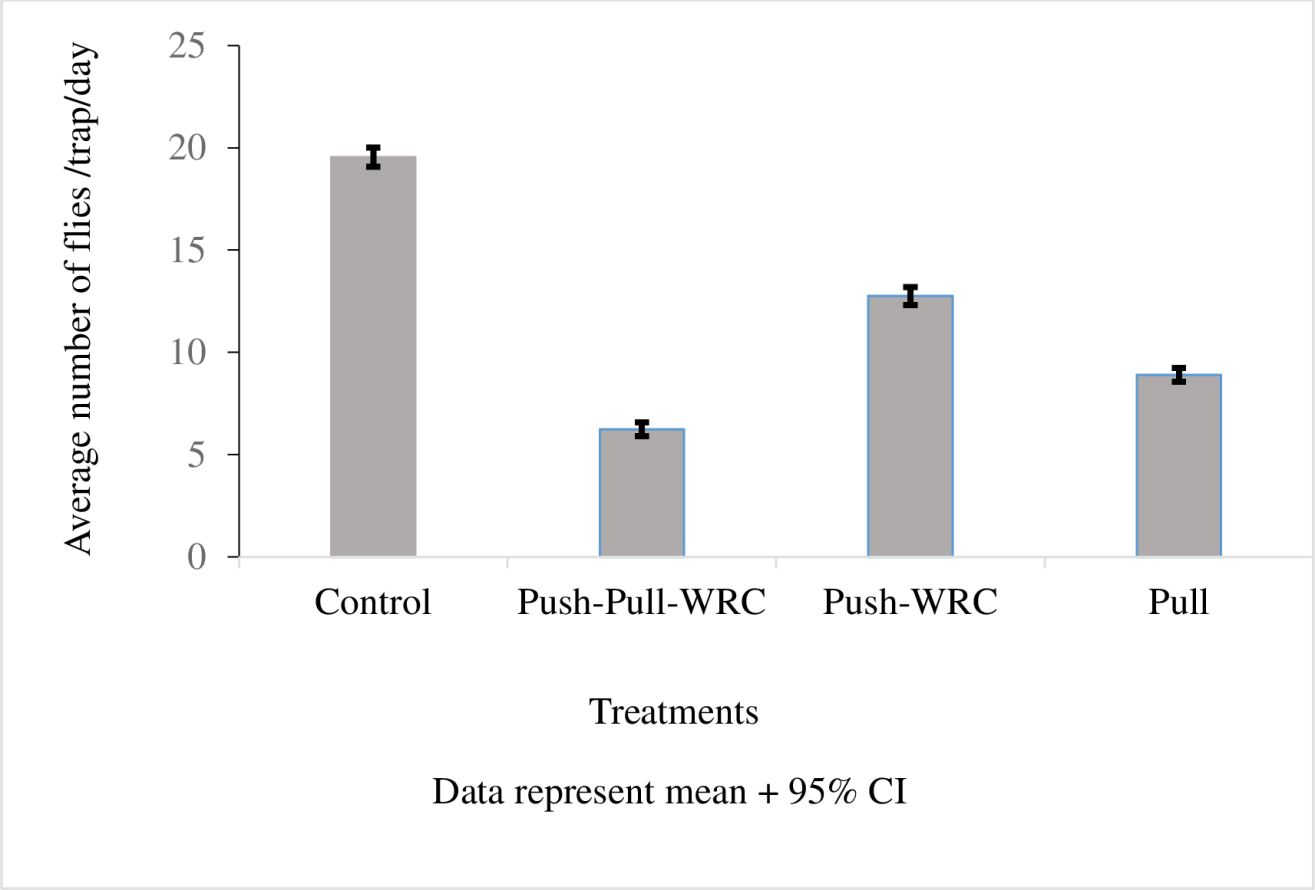
Average number of *G*. *pallidipes* flies caught per trap per day (apparent densities) with various tsetse repellent (WRC) treatments over the trial period.

Data were available on 68, 276 *G*. *pallidipes*. Compared to control and after adjusting for month of sampling there were, on average, significantly fewer fly catches in push-pull-WRC (RR = 0.33, 95%CI 0.30–0.36, P<0.0001), push-WRC (RR = 0.64, 95%CI 0.59–0.69, P<0.0001) and pull (RR = 0.46, 95%CI 0.42–0.49, P<0.0001) groups, with apparent densities being lowest amongst the push-pull-WRC treatment group. The results also indicated that on average, there were significant differences between push-pull-WRC and push-WRC (RR = 0.52, 95%CI 0.48–0.56; P<0.0001), push-pull-WRC and pull (RR = 0.73, 95%CI 0.67–0.79; P<0.0001), and between push-WRC and pull (RR = 1.40, 95%CI 1.29–1.52; P<0.0001) after controlling for the effect of time (Fig 7). The dispersion parameter was 1.15 (95%CI 1.10–1.20) significantly different from zero and hence we are justified in our negative binomial model rather than a Poisson model.

### Functioning of repellent dispensers

Overall, collars were put on 500 animals in the push-WRC and push-pull-WRC groups, most of which were males (59.3%). In total 5,740 visits/inspections were made to collar-wearing animals throughout the entire study period. In 4,355 of these inspections (i.e. 75.9%; 95% CI 74.74–76.97%), the dispensers were found to be working (push-pull-WRC 73.5%, and push-WRC 77.6%), whereas in 1,385 dispensers were defective. The reasons for the latter were (i) dry dispensers/ repellent leaked (32.6%; n = 452), (ii) tygon tubing dislodged from the nozzles (26.4%; n = 366), (iii) tubing torn or lost (24.8%; n = 344), and (iv) screw caps or entire dispenser missing (16.1%; n = 223). Thus, in our trial, significant disease reduction and associated impacts with the WRC have been achieved despite the prototype and artisan nature of the repellent dispensers and with only about 75% dispensers working during the field trials.

## Social-economic impact of tsetse repellent collars

Socio-economic impact of the tsetse repellent collars on farmers’ perceptions on effectiveness of the collars and on grazing patterns; impact on oxen owned, traction power and land cultivated; drug use and willingness to purchase the collars were assessed and established the social value of our approach.

### Farmers perceptions on the effectiveness of repellent collars and grazing patterns:

**Fig 8:**
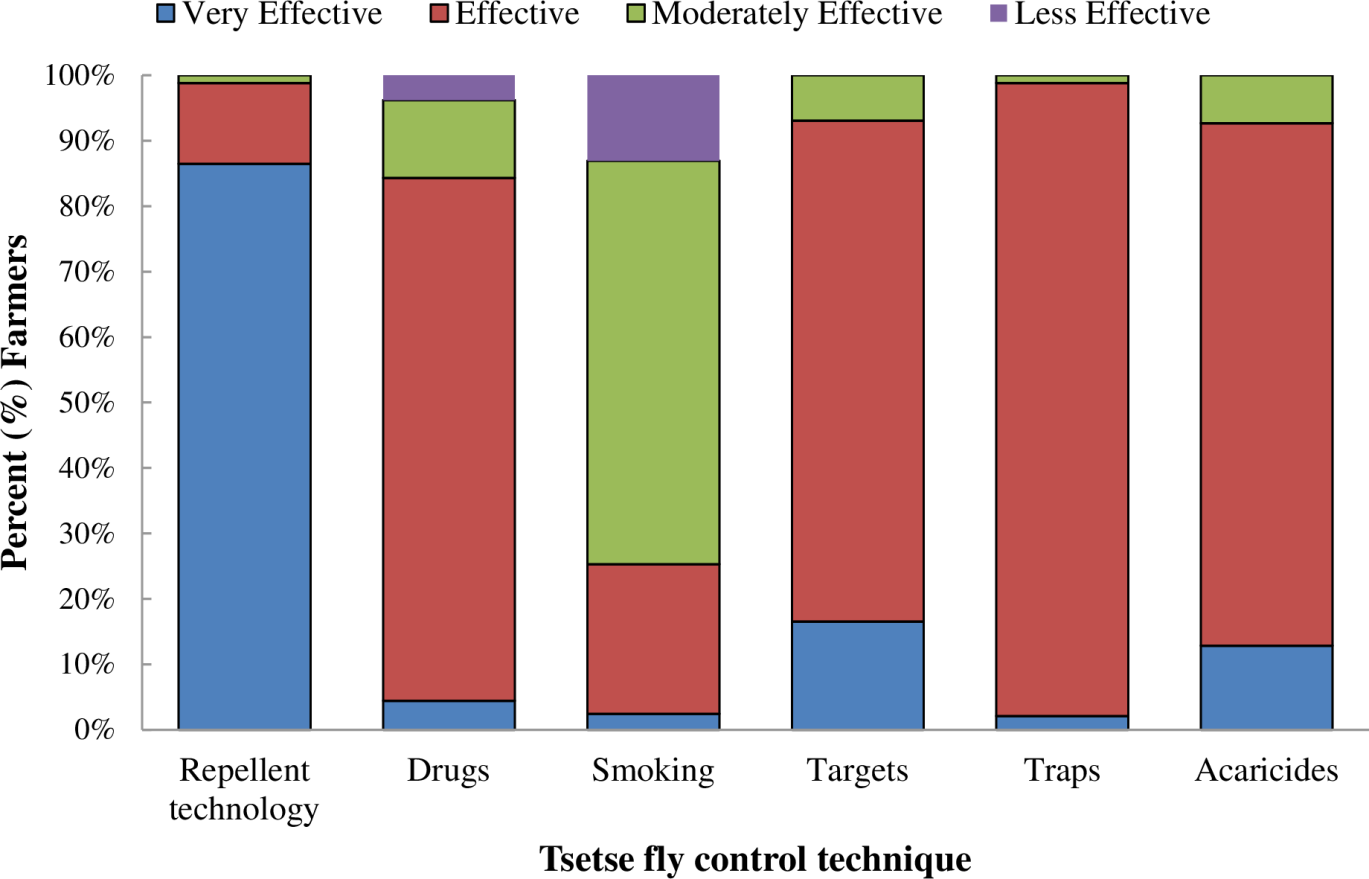
Farmers perceptions of the effectiveness of the new repellent collar technology.

**Fig 9:**
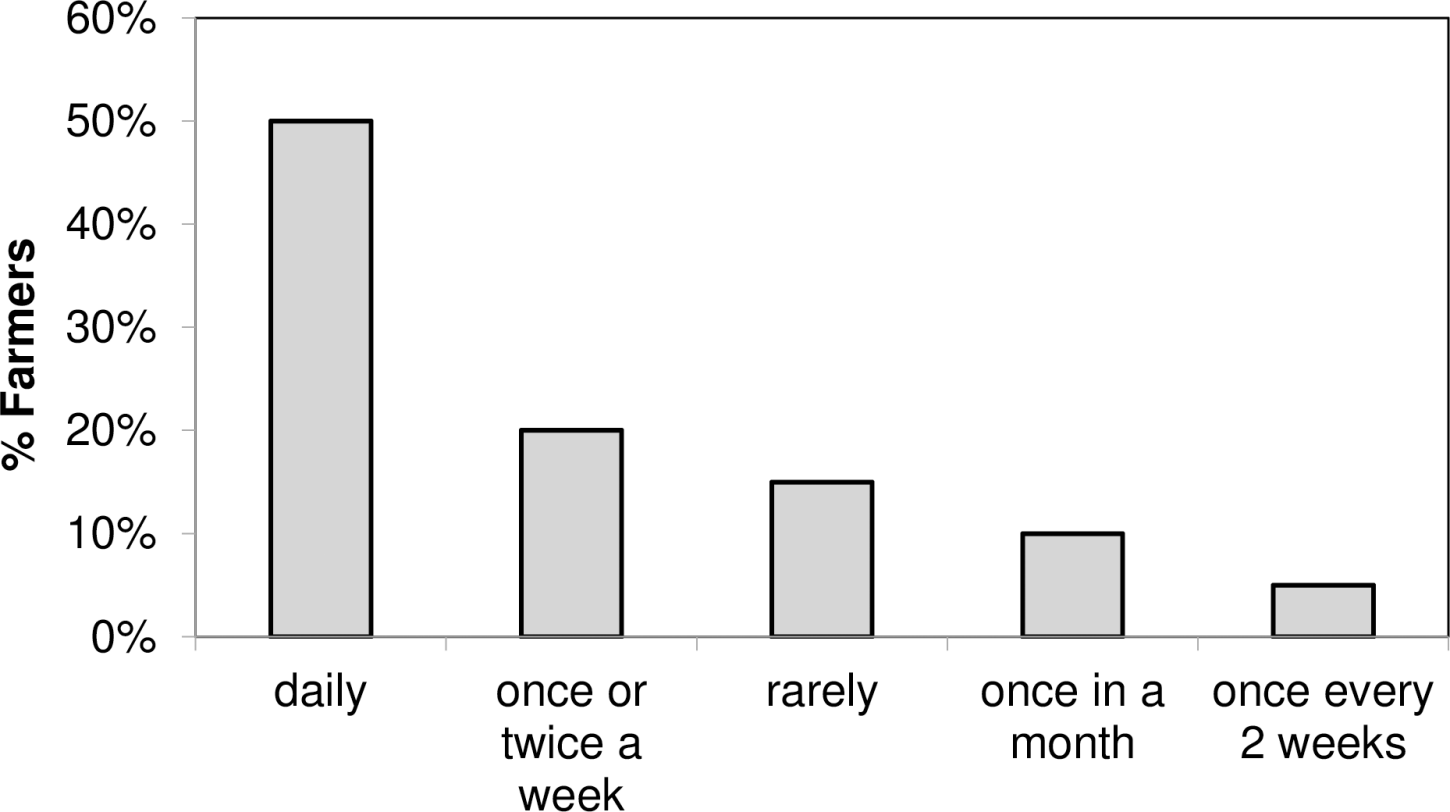
Non-participating farmers’ grazing practices that take advantage of cattle protected with repellent collars.

Majority (99%) of the farmers sampled (N = 178) rated the effectiveness of the repellent collars being between very effective (87%) and effective (12%) (Fig 8). Fifty-five percent of the non-participating farmers also found the technology to be very effective, and 46.5% of households (n = 43) that did not have access to repellent collars grazed their animals next to herds that had collars, taking advantage of the volatile nature of the WRC. Of these 50% grazed their animals next to their neighbors protected animals daily, while 20% of them grazed them once or twice per week (Fig 9). Fig 8 also shows farmers’ perceptions of other commonly used tsetse control technologies including the use of acaricides (alpha cypermethrin) for tick control. Among the farmers surveyed (n = 166), 75% mentioned that pyrethroids were infrequently used. Among, those who had used them, only 10% rated the acaricides as being very effective. The livestock keepers mentioned that the main problems with the acaricide dips were acute shortage of water during the dry seasons, very frequent dipping required especially during the rainy seasons, costs involved and resistance.

When asked which tsetse fly control technique they preferred the most, 81% (n = 166) of the respondents stated that they preferred the repellent technology more than any other techniques while 2% (N = 178) of the farmers preferred a combination of the repellent collars with targets, or traps or pour ons.

### Farmers’ perceptions on the impact of repellent collars on oxen owned, traction power and land cultivated for crop production:

Shimba Hills is a high-oxen-use area for ploughing, with 80% of the farmers using oxen for land preparation. Prior to the intervention 36.8% (n = 53) of the farmers hired oxen from low tsetse infested areas to plough their land while after the introduction of the repellent collars this was reduced by 66.3%. After the intervention, the number of farmers using their oxen for traction increased by 38.7% (before intervention 63.2% of farmers (n = 91) used their own oxen for ploughing while after the intervention the number increased to 87.6% (n = 141)).

The repellent collars also had a significant effect on the number of oxen owned per household in the study area (Chi Sq = 648, p<0.0001). Before the intervention, the average number of oxen per household were 2.2±0.39 (n = 40) while after the introduction of the repellent collars these increased to 3.8±0.32 –an increase of 72.7%. Overall, an increase in ownership of oxen had a significant effect (Chi Sq = 14148, p<0.001) on the land under crop production, increasing from 4.06±0.48 before the intervention to 7.04±0.59 after the intervention, constituting an increase of 73.4%. A significant difference in the increase of acres under cultivation was also recorded between treatments after the intervention (Chi Sq = 18.172, df = 3, p = 0.0004). A post-hoc test using Mann-Whitney tests with Bonferroni correction revealed that the number of acres under cultivation within the push-WRC differed significantly from the control (p = 0.0105) as did push-pull-WRC (p< 0.001). However, the pull treatment did not differ significantly from the control or the push-pull-WRC and push-WRC treatment. Overall, protected animals within the push-WRC and push-pull-WRC treatments ploughed 69% and 62% more land, respectively, compared to un-protected animals (control). In the area with targets alone (pull) oxen only ploughed 31% more land than those in the control block (Table 5) indicating that animals with repellent collars were healthier and had greater traction power. Increase in traction power of protected animals also influenced the number of acres tilled by hand per household per ploughing season recording a 69.1% reduction (1.01 ± 0.24 acres before the intervention and 0.31 ± 0.13 after the intervention).

**Table 5:** Number of acres ploughed by oxen in a ploughing season before and after the introduction of repellent collars. Means followed by the same letter in rows and columns are not significantly different.

Treatment	N	Before	After
Control	36	3.30 ± 0.33^a^	3.73 ± 0.30^a^
pull	34	3.79 ± 0.33^a^	4.87 ± 0.58^ab^
push–pull-WRC	31	4.34 ± 0.35^a^	6.04 ± 0.61^b^
push WRC	34	4.74 ± 0.53^a^	6.31 ± 0.61^b^

A sub-sample of 40 households indicated that after the introduction of the repellent collars, due to enhanced traction power of protected oxen, land allocated to cash crops (maize, sweet potatoes, beans, cow peas, tunguja—an Asian vegetable and passion fruits) increased on average 2x which contributed to the yield of these crops increasing 2.2x. Overall these farmers mentioned that in spite of some of this yield being used for household consumption the income from sale of these crops increased from US$ 429.27 to US$ 1056.75 per household/annually (an increase of 146%) and thereby significantly enhanced household income and food security after introduction of the repellent collars.

### Farmers’ perceptions on the impact of repellent collars on drug use

In Shimba Hills which is a high disease challenge area, farmers are totally dependent on drugs for control of nagana. Ninety-nine percent of the farmers sampled noticed a significant reduction in trypanocide use and related expenses after introduction of the repellent collars. Before the intervention, farmers in all blocks treated their animals at least 2.22 ± 0.20 times a month with trypanocides. After the intervention, the number of treatments per month significantly decreased to less than 1 treatment per month (0.91 ± 0.06), with some farmers reporting that it took more than a month to treat their animals (Chi Sq = 1085.5, df = 3, p<0.0001). This was a 58.1% reduction in number of trypanocide treatments in the entire study area.

The number of trypanocide treatments per month also significantly differed between treatment blocks after the introduction of the repellent collars (Chi Sq = 12.52, df = 3, p<0.0001). A post-hoc test using Mann-Whitney tests with Bonferroni correction revealed that the respondents from the push-pull-WRC treatment block recorded significantly lower (73.1% reduction) number of times they treated their cattle per month compared to any other treatment block (Table 6). Respondents with un-protected animals (control) which were also treated with drugs when found to be infected only recorded a 41.7% reduction on treatments of their animals (Table 6).

**Table 6:** Number of times per month respondents treated animals with trypanocides before and after the introduction of repellent collars. Means followed by the same letter in rows and columns are not significantly different (n = 40).

Treatment	Before	After	% Reduction
Control	1.8 ± 0.32^a^	1.0 ± 0.12^a^	41.7
push-WRC	2.5 ± 0.40^a^	1.0 ± 0.05^a^	62.0
pull	2.9 ± 0.34^a^	1.1 ± 0.14^a^	60.6
push-pull-WRC	1.5 ± 0.40^a^	0.4 ± 0.04^b^	73.1

Reduction in trypanocide usage is also reflected in the responses of the farmers on the amount of money they spend on them. On average, farmers incurred a monthly cost of US$ 10.68±1.65 on trypanocides to treat their herd before the intervention (US$ 1.78/cow assuming on average a herd contains 6 cows in the area). After the introduction of the repellent collars, there was a 77% reduction (US$ 0.69 per cow) in the amount of money farmers spent on trypanocides (Chi Sq = 12.52, df = 3, p = 0.005) which translates to a saving of US$1.09/cow/month and US$ 52.32 annually per herd (assuming 6 cows per herd and a maximum of 8 treatments per cow annually).

### Willingness to buy the repellant collars

Ninety-nine percent of the responding farmers (N = 178) in all the treatment blocks were willing to buy the repellent collars if they were available in the market. Sixty percent of the farmers were willing to invest in better breeds of their animals (from indigenous to exotic) if they could purchase the repellents.

### Costs of the repellent collars

Table 7, shows the current costs of the repellent collars compared to trypanocidal drugs which are the most common method of control. Presently, WRC and dispenser costs compare very favorably with trypanocidal drugs despite the prototype nature of the collars, with repellent collars, at currently US$ 2.7, being cheaper than drugs that require > 8 treatments/ year in high tsetse challenge areas like the SHGR. Moreover, it should be noted that the current metallic dispensers are a long-term and one-time investment and other than the tygon tubing (which should be replaced very six months) would last for a long time.

**Table 7:** Cost estimates of repellent collar/month/cow (A) compared to trypanocide use (B).

**(A) Cost of Repellent Dispenser and WRC (US$)**			(B) Treatment Costs (Chemotherapy/drugs)* (US$)		
**Materials**	**Per month**	**Annual**	**Trypanocide & Administration costs**	**Per month**	**8 treatments /year**
**Dispenser**	2.74	32.88	**Trypanocide**	1.78	14.24
**Tygon tubing**	0.19	2.28	**Veterinarian Fees**	3.33	26.64
**Canvas belt**	0.15	1.80	**Transport of Veterinarian**	0.93	7.44
**WRC**	0.87	10.44	**Syringe and Needle**	0.28	2.24
**Packaging & shipping**	0.04	0.48			
**Total**	**3.84**	**47.88**	**Total**	**6.32**	**50.56**

* Trypanocide use estimated at a maximum of 8 treatments/year/cow (B)

### Acceptance of repellent collars and establishment of a community based organization (CBO) to promote their use for tsetse control and exploitation of commercial opportunities provided

The very high acceptance of the repellent collars by the livestock keepers as being very effective and beneficial has motivated them to form and register their own community based organization (CBO) named ‘Kubo South Tsetse Control Self Help Group’ (P.O. Box 46–80407, Shimba Hills, Kwale, Kwale County, Kenya) with their own bank account. To date more than 1,200 farmers have registered as members of the CBO whose objective is to undertake integrated tsetse control using the repellent collars and to sell the dispensers and repellent compounds including servicing and maintenance of the dispensers once they are commercially available. So far, they have even collected about US$2,000 as seed money to buy the dispensers and the repellent compounds which is a positive sign towards adoption of the repellent collars and up-scaling of the repellent technology. The CBO is also in discussion with the local county government to subsidize the collars as part of the county government’s initiative to enhance livestock productivity in the county.

## Discussion

For the first time, we show how differential attraction of disease vectors to vertebrate animals can lead to identification of novel repellents which, when released from preferred host animals in the field using an innovative dispenser technology, can turn these animals into non-hosts, thus providing a new paradigm for disease control in livestock and also vector control. We demonstrate that semiochemicals identified from a non-preferred animal provide potent natural repellents that can be exploited to prevent contact between host and vector, thereby thwarting disease transmission (Fig 6, Table 2, and associated effects on Packed Cell Volume (PCV) Scores (Table 3) and weight gain of animals (Table 4) through behavioral modifications of the disease vector.

The huge (>80%) WRC-triggered reduction in disease incidence under natural tsetse challenge demonstrates the high repellent potency in pushing the flies away from cattle, and not absence of tsetse. Because nagana remains very high around SHGR (see baseline data [12]) because of continuous tsetse reinvasion into the trial sites, the work here has demonstrated a major step in control of vectors of livestock trypanosomosis. *G*. *m*. *morsitans* feed mainly on the belly and *G*. *pallidipes* mostly on the legs and, because in both species, the engorgements on the legs are concentrated mainly on the front legs [22], encircling the cow’s neck with repellent collars enabled the WRC to diffuse to the preferred feeding sites and thus provide individual protection *(‘cows wearing a waterbuck coat’)*.

In our studies, push-pull did not significantly enhance suppression of disease levels as the enormous WRC triggered reduction in disease incidence in cattle with repellent collars (push) was not significantly different from those in push-pull (Fig 6, Table 2) but the fly densities, were significantly lower in the push-pull compared to all other treatments (Fig 7) encouraging further development of this strategy. Significant weight gains in cattle with push-pull treatments compared to push (Table 4) also add to this encouragement. PCV levels, however, were as with disease levels significantly better in push as compared to push-pull treatments (Table 3). Push-pull incorporating these potent natural repellents could be further optimized for example, by using WRCs to ‘push’ the flies away and targets or a few animals with restricted insecticides applied on the legs and belly [22,23] as ‘pull’, to enhance disease and fly suppression in areas where isolated tsetse populations are present. Similarly, disease and fly reduction could also be improved with an optimized ‘pull’ in terms of strategic location and numbers of targets. Yet, in a dynamic system like the outskirts of SHGR where cattle and tsetse flies continually move and wild animals are also present, such parameters may be difficult to determine as was the case in our current trail where no synergistic or additive effect was observed with reduction in disease levels but only in reduction of fly densities. Perhaps the enormous disease reduction attained by push alone is the maximum that can be achieved. Moreover, since traps or targets suffer from fire, negative community behavior or reaction to them and perceptions associated with witchcraft [24, 25], it may be better to use insecticide treated livestock as ‘pull’ [26]. Nonetheless, this first demonstration of the suitability of push-pull for livestock protection encourages wider use against haematophagous arthropod vectors [15,16]. Our trial also confirms results of previous studies (22) that female animals are less likely to be infected with trypanosome infections than males. In addition, trypanosome infections in black colored animals were significantly higher than lighter colored animals.

Information intensive technologies depend heavily on community understanding and participation right from the beginning [27–29]. Here, communities were involved from the onset and perceptions of farmers and pastoralists about the repellent technology were highly positive, with 99% of them rating it as very effective (Fig 8). A similar proportion want to purchase the collars to protect their animals and also prefer (>80%) them over traps or targets or insecticide treatment of their livestock. Interestingly, >55% of non-participating farmers also attest to the effectiveness of the repellent collars. Moreover, 47% of the households in the trial sites that did not have access to the repellent collars grazed their animals next to protected herds and thus benefitted as WRC ‘free riders’ (Fig 9). In fact, WRC constituents are volatile compounds perceived through olfactory receptors on the flies’ antennae and thus are true spatial repellents [30] which, when control-released from the dispensers, build a cloud around the animal that discourages tsetse flies from contacting the host. Our socio-economic studies thus demonstrate the social value of this approach, with farmers/ pastoralists also reporting that, in protected animals, the frequency of trypanocide treatments and their associated costs were reduced by >70%. The improved health of the WRC protected animals is also reflected in the enhanced traction power of oxen, with >65% more land ploughed than by unprotected animals. Increased traction power of WRC protected animals also had an effect on the land tilled by hand, which was reduced by 69%. In addition, improved health of oxen resulted in 73% increased ownership of oxen after introduction of the repellent collars. Consequently, more farmers (87.6%) used their own oxen for ploughing rather than hiring (reduced by 63%) them, as was common prior to the intervention. The overall effect was an increase of >73% land being cultivated for crop production, which resulted in 2x more land being allocated for growing cash crops (maize, sweet potatoes, beans, cow peas, tunguja—an Asian vegetable and passion fruits), an increase in yield of 2.2x of these crops and increased income from sales of these crops from US$ 429.27 (prior to the intervention) to US$ 1,056.75 per household/annually (an increase of 146%) after introduction of the repellent collars. Reduction in trypanocide usage contributed to an additional saving of about US$52.32 per household with collars. Thus, just from sales of enhanced crop productivity and reduction of trypanocide usage farmers income increased by US$ 1,109.07 per household annually after introduction of the repellent collars which significantly contributed to the increase in quality of people’s lives and improved food security. In the current study, however, to fully quantify in economic terms other parameters like increase in ownership of oxen by 73%, reduction of hiring of oxen for ploughing by 66%, reduction in land tilled by hand by 69%, increase in selling price of protected animals (bulls by 26%), was not fully possible and such a detailed study needs to be undertaken to establish the full economic impact of the repellent collars. Our work thus supports the conclusion of Shaw *et al*., [31] that potential benefits of controlling tsetse and trypanosomiasis in East Africa are very high and the greatest potential benefits accrue due to the importance of animal traction with its contribution to crop production and in various roles in livelihood sustainability such as household income generation, cattle sales and social capital. WRC technology uptake, alone or in combination with other technologies, however, will depend on cost-effectiveness. Presently, WRC and dispenser costs compare very favorably with trypanocidal drugs despite the prototype nature of the collars, with repellent collars, at currently US$ 2.7, being cheaper than drugs that require > 8 treatments/ year in high tsetse challenge areas like SHGR area (Table 7). Thus, our socio-economic data clearly indicate that the adoption potential of the repellent collars is extremely high and the establishment of a CBO by the farmers to promote the use of the repellent collars for integrated control and to exploit them for commercialization attests to this. In this respect, our bottom up approach with the communities and their involvement right from the inception of the trials, very regular dialogue and feedback provided to the farmers on the results and progress of the project and the continuous feedback received from them on the performance of the collars has significantly enhanced the acceptance of the collars. Effective planning and budgeting for community involvement in all stages of the entire validation trail in our project coupled with the effectiveness and beneficial impact of the repellent collars have also significantly contributed to the very high acceptance of the repellent collars. Moreover, the potent repellent collars are private goods with benefits extending only to the livestock keepers using them to protect their cattle and thus chances of livestock keepers using them in a sustainable way for tsetse control are much higher. Unlike, traps or targets which are public goods dependent on entire communities and geographic areas for sustainability.

**Fig 10:**
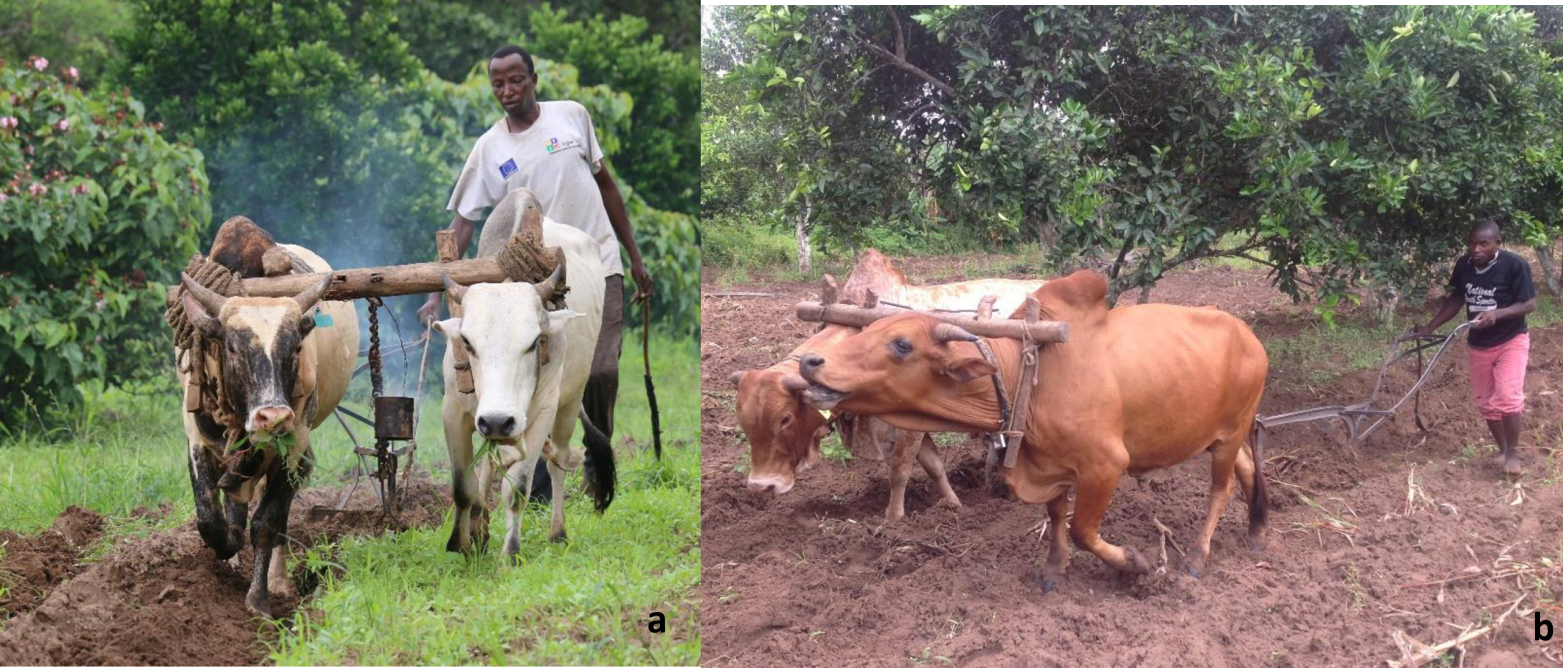
Farmer ploughing land by generating wood smoke (a) or using repellent collars (b) to protect oxen from tsetse flies. (Picture R.K. Saini).

Previous attempts using a synthetic repellent, 2-methoxy-4-methylphenol, for tsetse control failed because of design flaws of the dispensers [17, 32]. Our novel devices (Figs 3 and 4), however, are more robust (only <25% failed despite huge wear and tear in the herds) and when full to capacity, can provide ~four months of protection to an animal, provided no leakage or brakeage occurs. Incidentally, 2-methoxy-4-methylphenol [17] has been found to be a natural component of wood smoke which halved trap catches and reduced by ~70% the numbers of *G*. *pallidipes* and *G*. *m*. *morsitans* attracted to kraals [33], raising the possibility of developing a ‘synthetic smoke’ free of the health hazards associated with real smoke. In our trial, however, even though smoke has been used traditionally to protect cattle from tsetse farmers’ perception of smoke were not positive, with <20% of the respondents rating smoke effective (Figs 8 and 10). Our WRC may be one such effective synthetic combination for protecting people and their livestock from tsetse and other arthropod pathogen vectors attracted to cattle. Moreover, the response of vectors to compounds from related non-host species may be of more adaptive value in their behavioral ecology than their response to botanically derived repellents [34]. The high performance of the WRCs is likely to relate to the potential role of these compounds as stress cues associated with traits making prospective hosts unsuitable [34, 35]. Added advantages include minimum maintenance and no harmful effects on environmental, human or other animal health as the repellent compounds are natural products already present in tsetse habitats and importantly, also drastically reduce the reliance of livestock keepers and pastoralists in Africa on trypanocides. In addition, the immediate advantages of modifying vector behavior that results in movement away from a livestock/human host is a delayed or diminished development in the emergence of resistance to conventional insecticide resistance by minimizing the intensity of selection pressure from contact-mediated toxicity mechanisms as well as the potential of reduction of toxic effects to hosts and non-target organisms. If, however, the use of the current WRC is extended to a large proportion of at risk cattle, then resistance may develop over time. However, because the avoidance of unsuitable hosts is an important evolutionary advantage to the tsetse flies then other cues must substitute those in the WRC. Rather than in the case of insecticides where entirely new toxicophores may be needed to overcome resistance, here we would merely need to identify the new cues being used by the insect which could be readily done by the analytical methods, e.g. GC-EAG, used in the original identification of the WRC. In this respect, ongoing work by us to identify new cues from other non-preferred animals like zebra may also become useful. This argument with literature evidence has been expanded recently by Pickett & Khan [36].

The efficacy of non-host derived semio-chemicals like the WRC in addition to their efficiency in protecting cattle may also have implications for the control of the spread of sleeping sickness. The trypanosomes causing sleeping sickness are usually found at infection rates of 0.01% [37] and in low densities of tsetse populations found in these foci [38] repellents are likely to be more effective [33]. Just as WRC reduces the probability of tsetse entering and/or remaining in a trap [11] and also substantially reduces flies encountering a cow, the WRC may also protect man from tsetse and also deter them from entering houses or vehicles and thus reduce sleeping sickness further (the repellent dispensers do not have to be only on the neck of a cow but can be placed outside a dwelling). The WRC is currently being evaluated for vectors of human sleeping sickness (riverine tsetse common in West and Central Africa) with encouraging results.

This large-scale study, involving 1,100 cattle, demonstrates effective deployment of repellent-based technology to protect cattle from tsetse and associated nagana in SSA, with the advantage of drastic reduction in reliance on trypanocides. It also indicates how cost-effective technologies like the tsetse repellent technology impact livestock productivity and subsequently household income, labor and food security.

The development of our novel dispenser device now requires further improvement, in partnerships with the private sector for mass production and registration of the WRC formulation to allow for their use in tsetse infested countries in SSA. In Kenya, the registration of the WRC compounds by the Pest Control Products Board is already in the pipeline. Once fully optimized, the primary beneficiaries of this technology will be the often-marginalized pastoralists and small holder mixed farming practitioners of the semi-arid and sub-arid areas of Africa, who are recognized as amongst the poorest people in the world. For poor nomadic communities, this offers a mobile technology in which the animal wears the collar wherever it goes.

## Supporting information

S1 File Social economic evaluation of the tsetse repellent collars technology among participating and control herds in Shimba Hills in the coast of Kenya–detailed household questionnaire. (DOCX)

S2 File Social Economic Evaluation of the tsetse repellent collars technology among participating and control herds in Shimba Hills in the coast of Kenya–supplementary economic focused questionnaire. (DOCX)
