# Supplementary material for: Protecting cows in small holder farms in East Africa from tsetse flies by mimicking the odor profile of a non-host bovid
Source: PLoS Negl Trop Dis. 2017 Oct 17;11(10):e0005977. doi: 10.1371/journal.pntd.0005977 (PMC5659797; doi:10.1371/journal.pntd.0005977)
Supplement: S1 File — (DOCX) [file pntd.0005977.s001.docx]

# S1 File

# Social Economic Evaluation of the tsetse repellent collars technology among participating and control herds in Shimba Hills in the Coast of Kenya

## Detailed Household Questionnaire

### Section 00: Introduction and consent by main respondent

*Before the beginning of the interview read out the following paragraph and ensure that the respondent understands before asking for consent.*

*“Good morning/afternoon. We are coming from the International Centre for Insect Physiology and Ecology (ICIPE). We are conducting a survey looking at SOCIO ECONOMIC EVALUATION OF THE TSETSE REPELLENT COLLARS TECHNOLOGY, a product we have been testing in the Shimba Hills areas. We would like to ask you some questions. We would like to share some of this information widely in order that more people understand how the technology is working in this region and the issues that you face tsetse challenge and management.*

*Your name will not appear in any data that is made publicly available. The information you provide will be used purely for research purposes; your answers will not affect any benefits or subsidies you may receive now or in the future. Do you consent to be part of this study? You may withdraw from the study at any time and if there are questions that you would prefer not to answer then we respect your right not to answer them.*

*Contact Information: John Andoke of ICIPE:+254 720 050 702*

Q1. Has consent been given? (01.YES, 00.NO) [ __ __ ]

| Date of the interview  Q2-4. Start date (d/m/y)  Q5. Start Time (hr/min)  Q6. End Time (hr/min) | __ __ / __ __ / __ __ __ __  [ __ __: __ __ ]  [ __ __: __ __ ] |  |  |
| --- | --- | --- | --- |

## Section 01: Data Entry Information

|  | Name | Code | Date (d/m/y) | Signature |
| --- | --- | --- | --- | --- |
| Q1.1. Interviewer | _____________________ | [ __ __ ] | __ __ / __ __ / __ __ __ __ |  |
| Q1.2. Supervisor | _____________________ | [ __ __ ] | __ __ / __ __ / __ __ __ __ |  |
| Q1.3. Data entry clerk | _____________________ | [ __ __ ] | __ __ / __ __ / __ __ __ _ |  |

## Section 02: Geographical Information

| Q2.1. County  Q2.2. District  Q2.3. Location  Q2.4. Sub-Location  Q2.5. Village  Q2.6 ICIPE Block  Q2.7 Treatment in block | _______________________  _______________________  _______________________  _______________________  _______________________  ________________________  ________________________ | | [ __ __ ]  [ __ __ ]  [ __ __ ]  [ __ __ ]  [ __ __ ]  [__ __ ] | |  |  |
| --- | --- | --- | --- | --- | --- | --- |
| Q2.8. GPS coordinates (UTM) | | S: __________________ | | E: __________________ | | |
| Q2.9. Altitude (m) | | [ __ __ __ ● __ ] | |  | | |

## Section 03: Household Respondent and Type

*Ideal respondent: household head and/or spouse.*

| **Name of main respondent**  **Q3.1.** Person’s name (first)  **Q3.2.** Persons surname  **Q3.3.** Sex (00.Male, 01.Female)  **Q3.4.** Age (in Years)  **Q3.5.** Educational Level (Code A) | _______________________  _______________________  [ __ __ ]  [ __ __ ]  _____________________________ [ __ __ ]; |
| --- | --- |

| **Q3.6. Is the respondent the head of the household?** 01.YES, 00.NO | | | [ __ __ ] | |
| --- | --- | --- | --- | --- |
| **Q3.7. If NO, what is the relationship of main respondent to household head?** (Code B) | | | ______________ [ __ __ ] | |
| **Q3.8. Where is the household head?** (Code C) | | | ________________ [ __ __ ] | |
| **3.10 Household type.**  Put a **X** in situation that applies (Select only one) | [ _ ] Male headed, with one wife  [ _ ] Male headed with more than one wife;  [ _ ] Male headed, divorced, single or widowed;  [ _ ] Female headed, divorced, single or widowed;  [ _ ] Female headed, husband away, husband makes most household/agricultural decisions;  [ _ ] Female headed, husband away, wife makes most household/agricultural decision;  [ _ ] Child headed (age 16 or under)/Orphan;  [ _ ] Other, specify:______________ | | | |

| **Code A (EDUCATION)**  00. None  01. Standard 1  02. Standard 2  03. Standard 3  04. Standard 4  05. Standard 5  06. Standard 6  07. Standard 7  08. Standard 8 | 09. Form 1  10. Form 2  11. Form 3  12. Form 4  13. Craft/vocational  14. Certificate  15. Diploma  16. HND  17. University | **Code B (RELATIONSHIP TO HHH)**  00. Head 01. Spouse 02. Parent, 03. Child, 04. Grandchild  05. Other | **Code C (Where is HHH)**  01. Temporarily away from the house  02. Absent from home at least 6 months in a year  03. Other |
| --- | --- | --- | --- |

## Section 04: Demography

*We would like to know about you and your household. Kindly indicate the age group of the members of your household;*

| Q1. How many members of the household are in the age category below | Male | Female |
| --- | --- | --- |
| 0 years to 14 years |  | [ __ __ ] |
| 15 years to 64 years |  | [ __ __ ] |
| Over 64 years |  | [ __ __ ] |

## Section 05: Enterprises the household is involved in

*In this section we seek information on the main sources of income for your household*

| Q5.1. What are your household’s main sources of income? (Order the options from most important 1 to the least important 6)   1. Crop production and sales _____YES _________No________Rank_________ 2. Livestock production and sales _____YES _________No________Rank_________ 3. Formal employment _____YES _________No________Rank_________ 4. Informal business _____YES _________No________Rank_________ 5. Remittances _____YES _________No________Rank_________ 6. Others _____YES _________No________Rank_________ Please specify ________________________________________________________________________________ |  |
| --- | --- |
| Q5.2. Did you receive any cash and/or input (formal and informal) credit in 2012 for crop production (01.YES; 00.NO) | [ __ __] |
| Q5.5: If yes, from whom? ____________________________________________________ |  |
| Q5.7 If yes, what collateral was used? ____________________________________________ |  |
| Q5.6: How much credit was received? ______________________________________ |  |
| Q5.3. What was the purpose of obtaining the credit in 2012   1. Livestock production? (01.YES; 00.NO) 2. Crop production? (01.YES; 00.NO) 3. Payment of school fees? (01.YES; 00.NO) 4. Household consumption? (01.YES; 00.NO) 5. Others (Please specify) _______________________________________________________ | [ __ __]  [ __ __]  [ __ __]  [ __ __] |

## Section 06: The household cattle assets

*Indicate total cattle that you and your household own or manage currently in the farm where your homestead is.*

| Q6.1 Livestock (Name given to animal) | Q6.2. Bull or cow | Q6.3. Breed | Q6.4 Born or bought in | Q6.5. Age of the animal | Q6.6. Value of the animal if sold today | Q6.7 What would have been its value **before ICIPE** project |
| --- | --- | --- | --- | --- | --- | --- |
| 1 | [ __ __ __ __ ] |  | [ __ __ __ __ ] | [ __ __ __ __ ] | [ __ __ __ __ ] | [ __ __ __ __ ] |
| 2 | [ __ __ __ __ ] |  | [ __ __ __ __ ] | [ __ __ __ __ ] | [ __ __ __ __ ] | [ __ __ __ __ ] |
| 3 | [ __ __ __ __ ] |  | [ __ __ __ __ ] | [ __ __ __ __ ] | [ __ __ __ __ ] | [ __ __ __ __ ] |
| 4 | [ __ __ __ __ ] |  | [ __ __ __ __ ] | [ __ __ __ __ ] | [ __ __ __ __ ] | [ __ __ __ __ ] |
| 6 | [ __ __ __ __ ] |  | [ __ __ __ __ ] | [ __ __ __ __ ] | [ __ __ __ __ ] | [ __ __ __ __ ] |
| 7 | [ __ __ __ __ ] |  | [ __ __ __ __ ] | [ __ __ __ __ ] | [ __ __ __ __ ] | [ __ __ __ __ ] |
| 8 | [ __ __ __ __ ] |  | [ __ __ __ __ ] | [ __ __ __ __ ] | [ __ __ __ __ ] | [ __ __ __ __ ] |
| 9 | [ __ __ __ __ ] |  | [ __ __ __ __ ] | [ __ __ __ __ ] | [ __ __ __ __ ] | [ __ __ __ __ ] |
| 10 | [ __ __ __ __ ] |  | [ __ __ __ __ ] | [ __ __ __ __ ] | [ __ __ __ __ ] | [ __ __ __ __ ] |
| 11 | [ __ __ __ __ ] |  | [ __ __ __ __ ] | [ __ __ __ __ ] | [ __ __ __ __ ] | [ __ __ __ __ ] |
| 12 | [ __ __ __ __ ] |  | [ __ __ __ __ ] | [ __ __ __ __ ] | [ __ __ __ __ ] | [ __ __ __ __ ] |
| 13 | [ __ __ __ __ ] |  | [ __ __ __ __ ] | [ __ __ __ __ ] | [ __ __ __ __ ] | [ __ __ __ __ ] |
| 14 | [ __ __ __ __ ] |  | [ __ __ __ __ ] | [ __ __ __ __ ] | [ __ __ __ __ ] | [ __ __ __ __ ] |
| 15 | [ __ __ __ __ ] |  | [ __ __ __ __ ] | [ __ __ __ __ ] | [ __ __ __ __ ] | [ __ __ __ __ ] |
| 16 | [ __ __ __ __ ] |  | [ __ __ __ __ ] | [ __ __ __ __ ] | [ __ __ __ __ ] | [ __ __ __ __ ] |
| 17 | [ __ __ __ __ ] |  | [ __ __ __ __ ] | [ __ __ __ __ ] | [ __ __ __ __ ] | [ __ __ __ __ ] |
| 18 | [ __ __ __ __ ] |  | [ __ __ __ __ ] | [ __ __ __ __ ] | [ __ __ __ __ ] | [ __ __ __ __ ] |
| 19 | [ __ __ __ __ ] |  | [ __ __ __ __ ] | [ __ __ __ __ ] | [ __ __ __ __ ] | [ __ __ __ __ ] |
| 20 | [ __ __ __ __ ] |  | [ __ __ __ __ ] | [ __ __ __ __ ] | [ __ __ __ __ ] | [ __ __ __ __ ] |

*Indicate who owns what cattle in the family herd* ***currently.***

| Q6.7 Livestock | Q6.8. Number owned by male members | Q6.9. Number owned by female members | Q6.10 Number jointly owned | Q6.11. Number of livestock kept by household for others | Q6.12. Number of animals kept for the household by others away |
| --- | --- | --- | --- | --- | --- |
| Female cows (Exotic) | [ __ __ __ __ ] | [ __ __ __ __ ] | [ __ __ __ __ ] | [ __ __ __ __ ] | [ __ __ __ __ ] |
| Bulls (Exotic) | [ __ __ __ __ ] | [ __ __ __ __ ] | [ __ __ __ __ ] | [ __ __ __ __ ] | [ __ __ __ __ ] |
| Female cows (Indigenous) | [ __ __ __ __ ] | [ __ __ __ __ ] | [ __ __ __ __ ] | [ __ __ __ __ ] | [ __ __ __ __ ] |
| Bulls (Indigenous) | [ __ __ __ __ ] | [ __ __ __ __ ] | [ __ __ __ __ ] | [ __ __ __ __ ] | [ __ __ __ __ ] |
| Goats [all] | [ __ __ __ __ ] | [ __ __ __ __ ] | [ __ __ __ __ ] | [ __ __ __ __ ] | [ __ __ __ __ ] |
| Sheep [all] | [ __ __ __ __ ] | [ __ __ __ __ ] | [ __ __ __ __ ] | [ __ __ __ __ ] | [ __ __ __ __ ] |

*Indicate total cattle numbers that you and your household owned or managed* ***before*** *the ICIPE project was started in the village.*

| Q6.13 Livestock | Q6.14. Number owned by male members | Q6.15. Number owned by female members | Q6.16. Number jointly owned | Q6.17. Number of livestock kept for others | Q6.18. Number of animals kept for the household by others away | Q6.19 Are animals kept away cows or bulls |
| --- | --- | --- | --- | --- | --- | --- |
| Female cows (Exotic) | [ __ __ __ __ ] | [ __ __ __ __ ] | [ __ __ __ __ ] | [ __ __ __ __ ] | [ __ __ __ __ ] |  |
| Bulls (Exotic) | [ __ __ __ __ ] | [ __ __ __ __ ] | [ __ __ __ __ ] | [ __ __ __ __ ] | [ __ __ __ __ ] |  |
| Female cows (Indigenous) | [ __ __ __ __ ] | [ __ __ __ __ ] | [ __ __ __ __ ] | [ __ __ __ __ ] | [ __ __ __ __ ] |  |
| Bulls (Indigenous) | [ __ __ __ __ ] | [ __ __ __ __ ] | [ __ __ __ __ ] | [ __ __ __ __ ] | [ __ __ __ __ ] |  |
| Goats [all] | [ __ __ __ __ ] | [ __ __ __ __ ] | [ __ __ __ __ ] | [ __ __ __ __ ] | [ __ __ __ __ ] |  |
| Sheep [all] | [ __ __ __ __ ] | [ __ __ __ __ ] | [ __ __ __ __ ] | [ __ __ __ __ ] | [ __ __ __ __ ] |  |

*Indicate the differences you have noticed in the following aspect now and before the ICIPE project was started in the village.*

| **Calving rates** |  |  |
| --- | --- | --- |
| How many calves have been born in your herd for the last two years – July 2011 to July 2013? |  |  |
| Have all the calves survived? | Yes _____ No ___________ | If no, how many died? ___________ |
|  | When did the cows last calve (month, year)? | When was the previous calf born to this animal? (month, year) |
| Cow 1 | _________________________ | _____________________________ |
| Cow 2 | ________________________ | _____________________________ |
| Cow 3 | _________________________ | _____________________________ |
| Cow 4 | __________________________ | _____________________________ |
| What was the interval between calves **before** the start of the ICIPE project |  | |
| Cow 1 | ________________________ (Years) | |
| Cow 2 | ________________________ (Years) | |
| Cow 3 | ________________________ (Years) | |
| Cow 4 | ________________________ (Years) | |
| For a period of two years **before the ICIPE project**, how many calves were born in your herd? | __________________________ | |
| Did all the calves survive? | __________________________ | |
| If some died, how many died? | __________________________ | |

| **Death of animals** |  |  |
| --- | --- | --- |
| Are there any animals that have died in your herd in the last two years July 2011 – July 2013? | Yes ______ No _______ | If yes, how many? _________________ |
| Are there any animals that died in two years in your herd before the ICIPE project?? | Yes ______ No _______ | If yes, how many? _________________ |

| **Cattle Aborting** |  | **Any remarks** |
| --- | --- | --- |
| Have you ever experienced abortions among your cattle? | Yes ______ No _______ |  |
| How many times have you had abortions in your herd? |  |  |
| When was the last time you experienced an abortion | __________________ |  |

| **Area cultivated by oxen** |  |  |
| --- | --- | --- |
| Does your household plough your land using the oxen? | Yes ______ No _______ | If yes, how many acres? _________________ |
| Do you hire the oxen or use your own oxen? | hire ______ own _______ | If hired, how much do you pay? _________________ |
| How many days do you need to plough your land? | __________________ |  |
| Did your household plough your land using the oxen **before the ICIPE project**? | Yes ______ No _______ | If yes, how many acres? _________________ |
| Did you hire the oxen or used your own oxen before the ICIPE project?? | hire ______ own _______ | If hired, how much do you pay? _________________ |
| How many days did you need to plough your land before the ICIPE project?? |  |  |

| **Grazing of livestock** |  |  |
| --- | --- | --- |
| Where do you graze your livestock currently? | Own pasture farm ____________  Forest area ____________________  Near the park _________________  Neighbours plot _______________  Other (explain) ________________________________________  ________________________________________  ________________________________________ |  |
| What is the farthest point you can take your animals for grazing from your homestead? | ______________________________ | How many KMs away from home? _______________ |
| Where did you graze your animals before the ICIPE project started in the village? | Own pasture farm ____________________  Forest area ____________________________  Neighbours plot _______________________  Other (explain) ________________________________________  ________________________________________  ________________________________________ |  |
| What was the farthest point you could take your animals for grazing before the ICIPE project started in the village? | ___________________________________ | How many KMs away from home? _______________ |

| **Sale/buying of animal** | **What category did you sell** | **At what price per animal?** |
| --- | --- | --- |
| How many animals have you sold in the last 12 months?  _____________________ | - Bulls _______________ - Cows _______________ - Calves ______________ - Heifers ______________ - Steers ________________ |  |
| How many animals did you buy into your herd in the last 12 months?   - ___________________________ | - Bulls _______________ - Cows _______________ - Calves ______________ - Heifers ______________ - Steers ________________ |  |
| **Before the ICIPE project started** |  |  |
| How many animals did you sell in one year?  _____________________ | - Bulls _______________ - Cows _______________ - Calves ______________ - Heifers ______________ - Steers ________________ |  |
| How many animals did you buy into your herd in one year?   - ___________________________ | - Bulls _______________ - Cows _______________ - Calves ______________ - Heifers ______________ - Steers ________________ |  |

| **Milk production among the cows** | | |
| --- | --- | --- |
| How many cows are **currently** wet (giving milk) now in your herd? | ________________________ |  |
| How much milk do the cows give   - Cow 1 - Cow 2 - Cow 3 - Cow 4 | Morning milk  Litres ______________  Litres ______________  Litres ______________  Litres _______________ | Evening milk  Litres ___________  Litres ___________  Litres ___________  Litres ___________ |
| How do you use the milk  Sales  Gifts  Household consumption  Others  ___________________________  ____________________________  ____________________________ | **Morning milk**  Litres __________ @ ______________  Litres __________________  Litre ___________________  Litres __________________  Litres __________________  Litres __________________ | **Evening Milk**  Litres __________ @ ___________  Litres __________________  Litre ___________________  Litres __________________  Litres __________________  Litres __________________ |
| How many cows were giving milk in your herd **before** the ICIPE project started in the village? |  |  |
| How much milk were the cows giving before the ICIPE project started in the village   - Cow 1 - Cow 2 - Cow 3 | **Morning milk**  Litres ______________  Litres ______________  Litres ______________ | **Evening milk**  Litres ___________  Litres ___________  Litres ___________ |
| How much milk were you using ICIPE project started in the village for:  Sales  Gifts  Household consumption  Others  ___________________________  ____________________________  ____________________________ | **Morning milk**  Litres __________ @ ______________  Litres __________________  Litre ___________________  Litres __________________  Litres __________________  Litres __________________ | **Evening Milk**  Litres __________ @ ___________  Litres __________________  Litre ___________________  Litres __________________  Litres __________________  Litres __________________ |

## Section 07: Source of information on livestock production

In this section, we would like to ask you about how you obtain information for livestock production to support your household in different areas of livestock production.

|  | **Technology/ Issue** | **In the last 12 months, where did you get information about the following practices –** put an X in all the sources of information that apply | | | | | | |
| --- | --- | --- | --- | --- | --- | --- | --- | --- |
|  | **Q7.1 Appropriate livestock breed** | [ _ ]Did not get any information  [ _ ] Government extension service  [ _ ] Farmer Coop or groups | [ _ ] NGOs  [ _ ] Field days  [ _ ] Barazas | [ _ ] Seed traders/Agrovets  [ _ ] Neighbour/other farmers  [ _ ] ICIPE Staff | [ _ ] School  [ _ ] Newspaper  [ _ ] Radio | | [ _ ] TV  [ _ ] Cell phone  [ _ ] __________ | |
|  | **Q7.2 Tsetse management in the livestock herd** | [ _ ] Did not get any information  [ _ ] Government extension service  [ _ ] Farmer Coop or groups | [ _ ] NGOs  [ _ ] Field days  [ _ ] Barazas | [ _ ] Seed traders/Agrovets  [ _ ] Neighbour/other farmers  [ _ ] ICIPE staff | [ _ ] School  [ _ ] Newspaper  [ _ ] Radio | | [ _ ] TV  [ _ ] Cell phone  [ _ ] __________ | |
|  | **Q7.3 Other Livestock diseases** | [ _ ] Did not get any information  [ _ ] Government extension service  [ _ ] Farmer Coop or groups | [ _ ] NGOs  [ _ ] Field days  [ _ ] Barazas | [ _ ] Seed traders/Agrovets  [ _ ] Neighbour/other farmers  [ _ ] ICIPE staff | [ _ ] School  [ _ ] Newspaper  [ _ ] Radio | | [ _ ] TV  [ _ ] Cell phone  [ _ ] __________ | |
|  | **Q7.4 Livestock marketing information** | [ _ ] Did not get any information  [ _ ] Government extension service  [ _ ] Farmer Coop or groups | [ _ ] NGOs  [ _ ] Field days  [ _ ] Barazas | [ _ ] Seed traders/Agrovets  [ _ ] Neighbour/other farmers  [ _ ] ICIPE staff | [ _ ] School  [ _ ] Newspaper  [ _ ] Radio | | [ _ ] TV  [ _ ] Cell phone  [ _ ] __________ | |
| Q7.5. In the last 12 months, how many contacts (times) did you have with government extension agents? | | | | | | | [ __ __ __ ] | |
| Q7.6. In the last 12 months, how many contacts (times) did you have with non-governmental organizations? | | | | | | | [ __ __ __ ] | |
| Q7.8. In the last 12 months, did you actively seek advice on tsetse challenge ?01. YES; 00. NO | | | | | | | [ __ __ ] | |
| Q7.9 If yes, from whom did you seek information on tsetse challenge? __________________________________________________________ | | | | | | |  | |

## Section 08: Management of tsetse

*In this section, we are going to ask you about the tsetse challenge and how you have managed it in your livestock herd.*

| - 1. Do you know about the tsetse fly?   2. Is tsetse a problem for your cattle herd? | Yes ____ No ______  Yes ____ No ______ | |  |
| --- | --- | --- | --- |
| - 1. Do you control for tsetse in your cattle herd | Yes _____ No ______ | |  |
|  |  | |  |
| **Impregnated Targets** |  | |  |
| - 1. Do you know about the ‘Impregnated targets’ as a method of tsetse control? | Yes _______ No _________ | |  |
| - 1. Have you ever applied this method in your cattle herd?   2. If yes, when did you first use it?   3. If yes, are you still using it to date? | Yes ________ No _________  _______________________  Yes ______ No __________ | |  |
| - 1. Please indicate how you assess the effectiveness of the ‘impregnated targets’ as a tsetse control technology | Very effective _________  Effective _____________  Moderately effective ______  Less effective _________  Not effective _________ | |  |
| - 1. What characteristics of the impregnated targets technology do you like most?   _________________________________________________________________________  __________________________________________________________________________  __________________________________________________________________________  __________________________________________________________________________  __________________________________________________________________________ |  | |  |
| - 1. What characteristics of the impregnated targets technology didn’t you like?   _________________________________________________________________________  __________________________________________________________________________  __________________________________________________________________________  __________________________________________________________________________  __________________________________________________________________________ |  | |  |
| - 1. How much money do you need to apply the impregnated target on your cattle herd [for six months]?? | _________________________ | |  |
| - 1. Who pays for the impregnated targets for your household?      1. The government thro DVO _____________      2. A project yes _______________ No ___________      3. Which project _______________________________________________      4. A community group _______________________      5. One household member ___________________      6. Other (specify) ____________________________________ |  | |  |
| - 1. Are you goats/sheep protected from tsetse when you use this technology in your cows? | Yes ___________ No _________ | |  |
|  |  | |  |
| **Repellant Collars** |  | |  |
| - 1. Do you know about the ‘repellant collars’ as a method of tsetse control? | Yes _______ No _________ | |  |
| - 1. Have you ever applied this method in your cattle herd?   2. If yes, when did you first use it?   3. If yes, are you still using it to date? | Yes ________ No _________  _______________________  Yes ______ No __________ | |  |
| - 1. If you don’t have a repellant collar in your herd, do you know anyone in the neighbour-hood who has one?   2. Do you ever take your animals to graze next to the animals of that person?   3. If yes, how often      1. Daily _____________________      2. Once or twice a week ___________________________      3. Once every two weeks ____________________________      4. Once in a month ___________________________________      5. Rarely ___________________________________ | Yes ________ No ____________  Yes _________ no _____________ | |  |
| - 1. Please indicate how you assess the effectiveness of the ‘repellent collars’ as a tsetse control technology | Very effective _________  Effective _____________  Moderately effective ______  Less effective _________  Not effective _________ | |  |
| - 1. What characteristics of the repellant collars technology do you like most?   _________________________________________________________________________  __________________________________________________________________________  __________________________________________________________________________  __________________________________________________________________________ |  | |  |
| - 1. What characteristics of the repellent collars technology didn’t you like?   _________________________________________________________________________  __________________________________________________________________________  __________________________________________________________________________  __________________________________________________________________________ |  | |  |
| - 1. If you were to get the tsetse repellant collar from the market, would you be willing to buy it for your livestock herd?   2. please indicate the highest amount you would be willing to pay for the collar | Yes ______ No ________  Ksh ______________ |  |  |
| - 1. How many months would you prefer to stay before refilling the colour? |  |  |  |
| - 1. What improvements would you suggest for the collar?   ______________________________________________________________  _______________________________________________________________  _______________________________________________________________  ________________________________________________________________ |  |  |  |
| - 1. Where would you get the money to buy the tsetse repellant collar?   _____________________________________________________  _____________________________________________________ |  |  |  |
| - 1. If you keep indigenous animals, would you change to exotic breeds if you had access to enough collars? | Yes ______ No ________ |  |  |
| - 1. Are you goats/sheep protected from tsetse when you use this technology in your cows? | Yes ______ No ____________ | | |
|  |  | | |
| **Treating with drugs** |  | | |
| - 1. Do you know about the ‘drugs use’ as a method of management of disease caused by tsetse? | Yes _______ No _________ | | |
| - 1. Have you ever used drugs to treat your cattle? | Yes ________ No _________ | | |
| - 1. If yes, when did you first use it? | _______________________ | | |
| - 1. If yes, are you still using drugs to date? | Yes ______ No __________ | | |
| - 1. Which drugs do you use for treating you cattle | Preventive (red) __________  Curative (yellow) __________ | | |
| - 1. How much of the drugs do you currently use per animal every six month?      1. Preventive (red) ____________ mls      2. Curative (Yellow) ______________ mls | At what cost:  Preventive (red) __________ ksh  Curative (yellow) __________ ksh | | |
| - 1. How much of the drugs did you use per animal every six month before the ICIPE project?      1. Preventive (red) ____________ mls      2. Curative (Yellow) ______________ mls | At what cost:  Preventive (red) __________ ksh  Curative (yellow) __________ ksh | | |
| - 1. Please indicate how you assess the effectiveness of the ‘drugs’ as a management to control disease caused by tsetse | Very effective _________  Effective _____________  Moderately effective ______  Less effective _________  Not effective _________ | | |
| - 1. Does your household keep drugs for use ‘just in case’ something happens? | Yes _________ No ________ | | |
| - 1. How much drugs do you keep? | ___________________________ | | |
| - 1. What characteristics of the drugs technology do you like most?   _________________________________________________________________________  __________________________________________________________________________  __________________________________________________________________________  __________________________________________________________________________  __________________________________________________________________________ |  | | |
| - 1. What characteristics of the drugs technology didn’t you like?   _________________________________________________________________________  __________________________________________________________________________  __________________________________________________________________________  __________________________________________________________________________ |  | | |
| - 1. How much money do you need to apply the drugs on your herd [for six months]? | _________________________ | | |
| - 1. Who pays for the drugs for your household?      1. The government thro DVO _____________      2. A project yes _______________ No ___________      3. Which project _______________________________________________      4. A community group _______________________      5. One household member ___________________      6. Other (specify) ____________________________________ |  | | |
| - 1. Are you goats/sheep protected from tsetse when you use this technology in your cows? | Yes __________ No ___________ | | |
| **Smoking** |  | | |
| - 1. Do you know about the ‘smoking’ as a method of tsetse control? | Yes _______ No _________ | | |
| - 1. Have you ever applied this method in your livestock herd?   2. If yes, when did you first use it?   3. If yes, are you still using it to date?   4. What materials do you burn to control tsetse?   __________________________________________________________  __________________________________________________________ | Yes ________ No _________  _______________________  Yes ______ No __________ | | |
| - 1. Please indicate how you assess the effectiveness of the ‘smoking’ as a tsetse control technology | Very effective _________  Effective _____________  Moderately effective ______  Less effective _________  Not effective _________ | | |
| - 1. What characteristics of the smoking technology do you like most?   _________________________________________________________________________  __________________________________________________________________________  __________________________________________________________________________  __________________________________________________________________________  __________________________________________________________________________ |  | | |
| - 1. What characteristics of the smoking technology didn’t you like?   _________________________________________________________________________  __________________________________________________________________________  __________________________________________________________________________  __________________________________________________________________________  __________________________________________________________________________ |  | | |
| - 1. Are you goats/sheep protected from tsetse when you use this technology in your cows? | Yes ________ No ____________ | | |
|  |  | | |
| **Netted Zero grazing units** |  | | |
| - 1. Do you know about the ‘netted zero grazing unit’ as a method of tsetse control? | Yes _______ No _________ | | |
| - 1. Have you ever applied this method in your livestock herd?   2. If yes, when did you first use it?   3. If yes, are you still using it to date? | Yes ________ No _________  _______________________  Yes ______ No __________ | | |
| - 1. Please indicate how you assess the effectiveness of the ‘Netted zero grazing unit’ as a tsetse control technology | Very effective _________  Effective _____________  Moderately effective ______  Less effective _________  Not effective _________ | | |
| - 1. What characteristics of the netted zero grazing unit technology do you like most?   _________________________________________________________________________  __________________________________________________________________________  __________________________________________________________________________  __________________________________________________________________________  __________________________________________________________________________ |  | | |
| - 1. What characteristics of the netted zero grazing unit technology didn’t you like?   _________________________________________________________________________  __________________________________________________________________________  __________________________________________________________________________  __________________________________________________________________________  __________________________________________________________________________ |  | | |
| - 1. How much money do you need to build and maintain the netted unit your herd [for six months]? | _________________________ | | |
| - 1. Who pays for the building and maintaining the unit for your household?      1. The government thro DVO _____________      2. A project yes _______________ No ___________      3. Which project _______________________________________________      4. A community group _______________________      5. One household member ___________________      6. Other (specify) ____________________________________ |  | | |
| 10.88 Are you goats/sheep protected from tsetse when you use this technology in your cows? | Yes ______ No _______________ | | |
| INSECTICIDES TREATED SCREENS |  | | |
| - 1. Do you know about the ‘insecticide treated screens’ as a method of tsetse control? | Yes _______ No _________ | | |
| - 1. Have you ever used the insecticide treated screens as a method to manage tsetse?   2. If yes, when did you first use it?   3. If yes, are you still using it to date? | Yes ________ No _________  _______________________  Yes ______ No __________ | | |
| - 1. Please indicate how you assess the effectiveness of the ‘insecticides treated screens’ as a tsetse control technology | Very effective _________  Effective _____________  Moderately effective ______  Less effective _________  Not effective _________ | | |
| - 1. What characteristics of the ‘insecticides treated screens’ technology do you like most?   _________________________________________________________________________  __________________________________________________________________________  __________________________________________________________________________  __________________________________________________________________________  __________________________________________________________________________ |  | | |
| - 1. What characteristics of the ‘insecticide treated screens’ technology didn’t you like?   _________________________________________________________________________  __________________________________________________________________________  __________________________________________________________________________  __________________________________________________________________________  __________________________________________________________________________ |  | | |
| - 1. How much money do you need maintain the ‘insecticide treated screens’ in your herd [for six months]? | _________________________ | | |
| - 1. Who pays for the building and maintaining the unit for your household?      1. The government thro DVO _____________      2. A project yes _______________ No ___________      3. Which project _______________________________________________      4. A community group _______________________      5. One household member ___________________      6. Other (specify) ____________________________________ |  | | |
| 10.88 Are you goats/sheep protected from tsetse when you use this technology in your cows? | Yes ______ No _______________ | | |
|  |  | | |
| TRAPS |  | | |
| - 1. Do you know about the ‘TRAPS’ as a method of tsetse control? | Yes _______ No _________ | | |
| - 1. Have you ever used TRAPS as a method to manage tsetse?   2. If yes, when did you first use it?   3. If yes, are you still using it to date? | Yes ________ No _________  _______________________  Yes ______ No __________ | | |
| - 1. Please indicate how you assess the effectiveness of the ‘TRAPS’ as a tsetse control technology | Very effective _________  Effective _____________  Moderately effective ______  Less effective _________  Not effective _________ | | |
| - 1. What characteristics of the ‘TRAPS’ technology do you like most?   _________________________________________________________________________  __________________________________________________________________________  __________________________________________________________________________  __________________________________________________________________________  __________________________________________________________________________ |  | | |
| - 1. What characteristics of the TRAPS technology didn’t you like?   _________________________________________________________________________  __________________________________________________________________________  __________________________________________________________________________  __________________________________________________________________________  __________________________________________________________________________ |  | | |
| - 1. How much money do you need maintain the ‘TRAP’ in your herd [for six months]? | _________________________ | | |
| - 1. Who pays for the building and maintaining of TRAPs for your household?      1. The government thro DVO _____________      2. A project yes _______________ No ___________      3. Which project _______________________________________________      4. A community group _______________________      5. One household member ___________________      6. Other (specify) ____________________________________ |  | | |
| - 1. Are you goats/sheep protected from tsetse when you use this technology in your cows? | Yes ______ No _______________ | | |
|  |  | | |
| **‘POUR ONs’ Technology** |  | | |
| - 1. Do you know about the ‘pour ons’ as a method of tsetse control? | Yes _______ No _________ | | |
| - 1. Have you ever applied this method in your cattle herd?   2. If yes, when did you first use it?   3. If yes, are you still using it to date?   4. How frequently do you apply a new ‘pour on’ on your herd? | Yes ________ No _________  _______________________  Yes ______ No __________  _________________________ | | |
| - 1. Please indicate how you assess the effectiveness of the ‘pour ons’ as a tsetse control technology | Very effective _________  Effective _____________  Moderately effective ______  Less effective _________  Not effective _________ | | |
| - 1. What characteristics of the pour on technology do you like most?   _________________________________________________________________________  __________________________________________________________________________  __________________________________________________________________________  __________________________________________________________________________ |  | | |
| - 1. What characteristics of the pour on technology didn’t you like?   _________________________________________________________________________  __________________________________________________________________________  __________________________________________________________________________  __________________________________________________________________________ |  | | |
| - 1. How much money do you need to apply the pour ons on your herd [for six months]? | _________________________ | | |
| - 1. Who pays for the pour ons for your household?      1. The government thro DVO _____________      2. A project yes _______________ No ___________      3. Which project _______________________________________________      4. A community group _______________________      5. One household member ___________________      6. Other (specify) ____________________________________ |  | | |
| - 1. Are you goats/sheep protected from tsetse when you use this technology in your cows? | Yes __________ No __________ | | |
|  |  | | |
| **Acaricides in DIPs** |  | | |
| - 1. Do you know about the ‘dipping in acaricides’ as a method of tsetse control? | Yes _______ No _________ | | |
| - 1. Have you ever applied this method in your cattle herd?   2. If yes, when did you first use it?   3. If yes, are you still using it to date?   4. How often do you take your animals to the dip? | Yes ________ No _________  _______________________  Yes ______ No __________  _________________________ | | |
| - 1. Please indicate how you assess the effectiveness of the ‘dipping in acaricides’ as a tsetse control technology | Very effective _________  Effective _____________  Moderately effective ______  Less effective _________  Not effective _________ | | |
| - 1. What characteristics of the dipping in acaricides on technology do you like most?   _________________________________________________________________________  __________________________________________________________________________  __________________________________________________________________________  __________________________________________________________________________  __________________________________________________________________________ |  | | |
| - 1. What characteristics of the dip acaricides technology didn’t you like?   _________________________________________________________________________  __________________________________________________________________________  __________________________________________________________________________  __________________________________________________________________________  __________________________________________________________________________ |  | | |
| - 1. How much money do you need to apply the dip acaricides on your herd [for six months]?? | _________________________ | | |
| - 1. Who pays for the acaricides for your household?      1. The government thro DVO _____________      2. A project yes _______________ No ___________      3. Which project _______________________________________________      4. A community group _______________________      5. One household member ___________________      6. Other (specify) _____________________________________________ |  | | |
| - 1. Are your goats/sheep protected from tsetse when you use this technology in your cows? | Yes _________ no _____________ | | |
|  |  | | |
| **Acaricides applied using knapsack sprayer** |  | | |
| - 1. Do you know about ‘‘spraying acaricides with knapsack sprayer’’ as a method of tsetse control? | Yes _______ No _________ | | |
| - 1. Have you ever applied this method in your cattle herd?   2. If yes, when did you first use it?   3. If yes, are you still using it to date?   4. How often do you spray your animals? | Yes ________ No _________  _______________________  Yes ______ No __________  __________________________ | | |
| - 1. Please indicate how you assess the effectiveness of the ‘spraying acaricides with knapsack sprayer’ as a tsetse control technology | Very effective _________  Effective _____________  Moderately effective ______  Less effective _________  Not effective _________ | | |
| - 1. What characteristics of the ‘spraying acaricides with knapsack sprayer’ technology do you like most?   _________________________________________________________________________  __________________________________________________________________________  __________________________________________________________________________  __________________________________________________________________________  __________________________________________________________________________ |  | | |
| - 1. What characteristics of the ‘spraying acaricides with knapsack sprayer’ technology didn’t you like?   _________________________________________________________________________  __________________________________________________________________________  __________________________________________________________________________  __________________________________________________________________________  __________________________________________________________________________ |  | | |
| - 1. How much money do you need to ‘spray acaricides with knapsack sprayer’ [for six months]?? | _________________________ | | |
| - 1. Who pays for the acaricides for your household?      1. The government thro DVO _____________      2. A project yes _______________ No ___________      3. Which project _______________________________________________      4. A community group _______________________      5. One household member ___________________      6. Other (specify) _____________________________________________ |  | | |
| - 1. Are your goats/sheep protected from tsetse when you use this technology in your cows? | Yes _________ no _____________ | | |
|  |  | | |

*In this section, kindly indicate your most preferred method of tsetse control*

| - 1. Of the methods you have listed above, which is your most preferred method/technology for controlling tsetse?   _______________________________________________   - 1. Why is it your most preferred method?   _______________________________________________  _______________________________________________  _______________________________________________  _______________________________________________ |  |
| --- | --- |

| **If you had a choice between the following technologies, which of the two would you choose?** | **Choice** | **Why?** |
| --- | --- | --- |
| Collars or traps |  |  |
| Collars or impregnated targets |  |  |
| Collars or drugs for treatment |  |  |
| Collars or insect treated screens |  |  |
| Collars or traps |  |  |

*In this section, please indicate the costs of maintaining your cattle herd for* ***one year (12 Months)***

| **Cost item** (e.g. drugs, feeds, labour, vet consultations**,** etc) | **Number/Amount required** | **Cost per unit** |
| --- | --- | --- |
|  |  |  |
|  |  |  |
|  |  |  |
|  |  |  |
|  |  |  |
|  |  |  |

## *In this section, please indicate the benefits you get from your cattle herd for one year (12 months)*

| **Benefits** (eg milk, calves, manure, drought power, etc) | **Number/Amount obtained** | **Price per unit** |
| --- | --- | --- |
|  |  |  |
|  |  |  |
|  |  |  |
|  |  |  |
|  |  |  |
|  |  |  |
|  |  |  |
|  |  |  |
|  |  |  |

## Section 9: Land Holdings

| Land attribute | Parcel 1 | Parcel 2 | Parcel 3 | Parcel 4 |
| --- | --- | --- | --- | --- |
| **Size (acres)** |  |  |  |  |
| **Year Acquired** |  |  |  |  |
| **Mode of acquisition**  (1=Inherited, 2=bought, 3=given by individual, 4=allocated by govt, 5=care taking for another, 6=renting/sharecropping, 7=Borrowed, 8=others) |  |  |  |  |
| **Current status**  (1=mainly cultivated, 2=mainly pasture, 3=mainly fallow, 4=mainly forested, 5=mainly rented away, 6=given to another person, 7=others) |  |  |  |  |
| Do you have a **title deed** to the parcel (1=yes, 0=no) |  |  |  |  |
| If you have no title, are the boundaries officially demarcated? (1=yes, 2=no) |  |  |  |  |
| If you have no title, and boundaries are not officially demarcated, are they demarcated informally by village elders (1=yes, 2=no) |  |  |  |  |
| Do you and your extended family farm on this parcel of land (1=yes, 0=no) |  |  |  |  |
| Are you restricted as to the crops you can grow on this parcel of land (1=yes, 0=no) |  |  |  |  |
| If yes, By who? |  |  |  |  |
| Can you allocate this parcel of land to your children without restrictions? (1=yes, 0=no) |  |  |  |  |
