# Supplementary material for: Protecting cows in small holder farms in East Africa from tsetse flies by mimicking the odor profile of a non-host bovid
Source: PLoS Negl Trop Dis. 2017 Oct 17;11(10):e0005977. doi: 10.1371/journal.pntd.0005977 (PMC5659797; doi:10.1371/journal.pntd.0005977)
Supplement: S2 File — (DOCX) [file pntd.0005977.s002.docx]

**S2 File**

**Social Economic Evaluation of the tsetse repellent collars Technology among participating and control herds in Shimba Hills in the Coast of Kenya**

**Supplementary Economic Focused Questionnaire**

**Section 02: Geographical Information**

| Q2.1. County  Q2.2. District  Q2.3. Location  Q2.4. Sub-Location  Q2.5. Village  Q2.6 ICIPE Block  Q2.7 Treatment in block | _______________________  _______________________  _______________________  _______________________  _______________________  ________________________  ________________________ | | [ __ __ ]  [ __ __ ]  [ __ __ ]  [ __ __ ]  [ __ __ ]  [__ __ ] | |  |  |
| --- | --- | --- | --- | --- | --- | --- |
| Q2.8. GPS coordinates (UTM) | | S: __________________ | | E: __________________ | | |
| Q2.9. Altitude (m) | | [ __ __ __ ● __ ] | |  | | |

**Section 03: Changes in land under plough**

Kindly indicate the changes that you have had on your farm since you started participating in the ICIPE project

|  | **Before the ICIPE project** | **After the ICIPE Project** |
| --- | --- | --- |
| 3.1 Number of oxen owned |  |  |
| 3.2 Number of acres under crop cultivation |  |  |
| 3.3 Number of acres ploughed by hand |  |  |
| 3.4 Number of acres ploughed by oxen |  |  |
| 3.5 Amount of money used to pay for oxen to plough the land |  |  |
| 3.6 The type of crops grown on the land  1  2  3  4  5  6  7  8  9  10  11  12 | **Before the ICIPE project**  Crop type ……acres under crop  ____________________________  ____________________________  ____________________________  ____________________________  ____________________________  ____________________________  ____________________________  ____________________________  ____________________________  ____________________________  ____________________________  ____________________________ | **After the ICIPE project**  Crop type ……acres under crop  ____________________________  ____________________________  ____________________________  ____________________________  ____________________________  ____________________________  ____________________________  ____________________________  ____________________________  ____________________________  ____________________________  ____________________________ |
| 3.6 For each crop type grown, indicate the yields obtained  1  2  3  4  5  6  7  8  9  10  11  12 | **Before the ICIPE project**  Crop type ……….units of yield  ____________________________  ____________________________  ____________________________  ____________________________  ____________________________  ____________________________  ____________________________  ____________________________  ____________________________  ____________________________  ____________________________  ____________________________ | **After the ICIPE project**  Crop type ……units of yield  ____________________________  ____________________________  ____________________________  ____________________________  ____________________________  ____________________________  ____________________________  ____________________________  ____________________________  ____________________________  ____________________________  ____________________________ |

**Section 04: Changes in INCOME on the farm**

|  | **Before the ICIPE project** | **After the ICIPE Project** |
| --- | --- | --- |
| 4.1 Do you sell crop produce on your farmer? | Yes ____________________  No _____________________ | Yes ____________________  No _____________________ |
| 3.6 Please indicate the crop produce that you sell on the farm (crop types)  1  2  3  4  5  6  7  8  9  10  11  12 | **Before the ICIPE project**  Amount sold……Units  ____________________________  ____________________________  ____________________________  ____________________________  ____________________________  ____________________________  ____________________________  ____________________________  ____________________________  ____________________________  ____________________________  ____________________________ | **After the ICIPE project**  Amount sold…….Units  ____________________________  ____________________________  ____________________________  ____________________________  ____________________________  ____________________________  ____________________________  ____________________________  ____________________________  ____________________________  ____________________________  ____________________________ |
| 3.6 For each crop type sold, please indicate the market where you sell currently and the prices obtained (crop type)  1  2  3  4  5  6  7  8  9  10  11  12 | **Market where sold**  ____________________________  ____________________________  ____________________________  ____________________________  ____________________________  ____________________________  ____________________________  ____________________________  ____________________________  ____________________________  ____________________________  ____________________________ | **Prices per unit**  ____________________________  ____________________________  ____________________________  ____________________________  ____________________________  ____________________________  ____________________________  ____________________________  ____________________________  ____________________________  ____________________________  ____________________________ |
|  |  |  |

**Section 05: Utilization of income**

5.1 For the income generated at the farm, please indicate what you did with the money in the last two season

| Use of money generated from the farm | Amount invested in that use | Who in the household benefited (head of household, wife, boy child, female child, all) |
| --- | --- | --- |
| 1. |  |  |
| 2 |  |  |
| 3. |  |  |
| 4. |  |  |
| 5. |  |  |
| 6. |  |  |
| 7. |  |  |
| 8. |  |  |
| 9. |  |  |
| 10. |  |  |

**Section 06: Household food security**

6.1. In the last 12 months, were there months in which you did not have enough food to meet your family's needs?01. Yes, 00. No [ __ __ ]

6.2. If yes, which were the months in the last 12 months in which you did not have enough food to meet your family's needs?

| Aug | Sep | Oct | Nov | Dec | Jan | Feb | Mar | Apr | May | Jun | Jul | Total |
| --- | --- | --- | --- | --- | --- | --- | --- | --- | --- | --- | --- | --- |
| [ __ ] | [ __ ] | [ __ ] | [ __ ] | [ __ ] | [ __ ] | [ __ ] | [ __ ] | [ __ ] | [ __ ] | [ __ ] | [ __ ] | [ __ __ ] |

6.3 12 months before the start of the ICIPE Project, were there months in which you did not have enough food to meet your family's needs? 01. Yes, 00. No [ __ __ ]

6.4 If yes, which were the months in which you did not have enough food to meet your family's needs before the ICIPE project?

| Aug | Sep | Oct | Nov | Dec | Jan | Feb | Mar | Apr | May | Jun | Jul | Total |
| --- | --- | --- | --- | --- | --- | --- | --- | --- | --- | --- | --- | --- |
| [ __ ] | [ __ ] | [ __ ] | [ __ ] | [ __ ] | [ __ ] | [ __ ] | [ __ ] | [ __ ] | [ __ ] | [ __ ] | [ __ ] | [ __ __ ] |

6.5 **Which of the following coping mechanisms against food shortage was used by your household before the ICIPE Project ?**Put an **X** to all that applies

| **Coping mechanisms** | |  | **Coping mechanisms** | |
| --- | --- | --- | --- | --- |
| **[ _ ]**  **[ _ ]**  **[ _ ]**  **[ _ ]**  **[ _ ]**  **[ _ ]**  **[ _ ]** | Reduced frequency of food intake  Withdrawing children from school  Reducing other expenditure  Selling small animals  Selling cattle  Selling farm equipment  Selling other assets |  | **[ _ ]**  **[ _ ]**  **[ _ ]**  **[ _ ]**  **[ _ ]**  **[ _ ]**  **[ _ ]** | Working more off-farm  Working at Food-for-Work  Receiving food aid  Receive gift from relatives  Consume seeds of next season  Harvest wild fruits  Other: ___________________ |

6.6 **Which of the following coping mechanisms against food shortage was used by your household AFTER the ICIPE Project ?**Put an **X** to all that applies

| **Coping mechanisms** | |  | **Coping mechanisms** | |
| --- | --- | --- | --- | --- |
| **[ _ ]**  **[ _ ]**  **[ _ ]**  **[ _ ]**  **[ _ ]**  **[ _ ]**  **[ _ ]** | Reduced frequency of food intake  Withdrawing children from school  Reducing other expenditure  Selling small animals  Selling cattle  Selling farm equipment  Selling other assets |  | **[ _ ]**  **[ _ ]**  **[ _ ]**  **[ _ ]**  **[ _ ]**  **[ _ ]**  **[ _ ]** | Working more off-farm  Working at Food-for-Work  Receiving food aid  Receive gift from relatives  Consume seeds of next season  Harvest wild fruits  Other: ___________________ |

6.7 **For each of the following questions, consider what has happened in the month before the last harvest.** Select one answer with a X

|  | **Never**  **(0 times)** | **Rarely**  **(1-2 times)** | **Sometimes (3-10 times)** | **Often (> 10 times)** |
| --- | --- | --- | --- | --- |
| **Q43.** Did you **worry** that your household would **not have enough food**? | 1. [ _ ] | 2. [ _ ] | 3. [ _ ] | 4. [ _ ] |
| **Q44.** Were you or any household member **not able to eat** the kinds of **foods you preferred**? | 1. [ _ ] | 2. [ _ ] | 3. [ _ ] | 4. [ _ ] |
| **Q45.** Did you or any household member eat **a limited variety of foods**? | 1. [ _ ] | 2. [ _ ] | 3. [ _ ] | 4. [ _ ] |
| **Q46.** Did you or any household member eat food that **you did not want to eat**? | 1. [ _ ] | 2. [ _ ] | 3. [ _ ] | 4. [ _ ] |
| **Q47.** Did you or any household member **eat a smaller meal than you felt you needed**? | 1. [ _ ] | 2. [ _ ] | 3. [ _ ] | 4. [ _ ] |
| **Q48.** Did you or any other household member **eat fewer meals in a day**? | 1. [ _ ] | 2. [ _ ] | 3. [ _ ] | 4. [ _ ] |
| **Q49.** Was there ever no food at all in your household? | 1. [ _ ] | 2. [ _ ] | 3. [ _ ] | 4. [ _ ] |
| **Q50.** Did you or any household member **go to sleep at night hungry**? | 1. [ _ ] | 2. [ _ ] | 3. [ _ ] | 4. [ _ ] |
| **Q51.** Did you or any household member **go a whole day without eating anything**? | 1. [ _ ] | 2. [ _ ] | 3. [ _ ] | 4. [ _ ] |

6.7 **For each of the following questions, consider what has happened in the months before a harvest (before the ICIPE project) s**elect one answer with a X

|  | **Never**  **(0 times)** | **Rarely**  **(1-2 times)** | **Sometimes (3-10 times)** | **Often (> 10 times)** |
| --- | --- | --- | --- | --- |
| **Q43.** Did you **worry** that your household would **not have enough food**? | 1. [ _ ] | 2. [ _ ] | 3. [ _ ] | 4. [ _ ] |
| **Q44.** Were you or any household member **not able to eat** the kinds of **foods you preferred**? | 1. [ _ ] | 2. [ _ ] | 3. [ _ ] | 4. [ _ ] |
| **Q45.** Did you or any household member eat **a limited variety of foods**? | 1. [ _ ] | 2. [ _ ] | 3. [ _ ] | 4. [ _ ] |
| **Q46.** Did you or any household member eat food that **you did not want to eat**? | 1. [ _ ] | 2. [ _ ] | 3. [ _ ] | 4. [ _ ] |
| **Q47.** Did you or any household member **eat a smaller meal than you felt you needed**? | 1. [ _ ] | 2. [ _ ] | 3. [ _ ] | 4. [ _ ] |
| **Q48.** Did you or any other household member **eat fewer meals in a day**? | 1. [ _ ] | 2. [ _ ] | 3. [ _ ] | 4. [ _ ] |
| **Q49.** Was there ever no food at all in your household? | 1. [ _ ] | 2. [ _ ] | 3. [ _ ] | 4. [ _ ] |
| **Q50.** Did you or any household member **go to sleep at night hungry**? | 1. [ _ ] | 2. [ _ ] | 3. [ _ ] | 4. [ _ ] |
| **Q51.** Did you or any household member **go a whole day without eating anything**? | 1. [ _ ] | 2. [ _ ] | 3. [ _ ] | 4. [ _ ] |
